# Upcycling of the Used Cigarette Butt Filters through Pyrolysis Process: Detailed Kinetic Mechanism with Bio-Char Characterization

**DOI:** 10.3390/polym15143054

**Published:** 2023-07-15

**Authors:** Bojan Janković, Marija Kojić, Milena Milošević, Milena Rosić, Hadi Waisi, Bojana Božilović, Nebojša Manić, Vladimir Dodevski

**Affiliations:** 1 Department of Physical Chemistry, “Vinča” Institute of Nuclear Sciences—National Institute of the Republic of Serbia, University of Belgrade, Mike Petrovića Alasa 12-14, 11001 Belgrade, Serbia; bojan.jankovic@vinca.rs; 2 Department of Radiation Chemistry and Physics, “Vinča” Institute of Nuclear Sciences—National Institute of the Republic of Serbia, University of Belgrade, Mike Petrovića Alasa 12-14, 11001 Belgrade, Serbia; marija.kojic@vinca.rs; 3 Institute of Chemistry, Technology and Metallurgy—National Institute of the Republic of Serbia, University of Belgrade, Njegoševa 12, 11000 Belgrade, Serbia; milena.milosevic@ihtm.bg.ac.rs; 4 Department of Material Science, “Vinča” Institute of Nuclear Sciences—National Institute of the Republic of Serbia, University of Belgrade, Mike Petrovića Alasa 12-14, 11001 Belgrade, Serbia; mrosic@vinca.rs; 5 Faculty of Ecology and Environmental Protection, University UNION-Nikola Tesla, Cara Dušana 62-64, 11000 Belgrade, Serbia; hadiwaisi@yahoo.com (H.W.); bozilovicbojana88@gmail.com (B.B.); 6 Institute of General and Physical Chemistry, University of Belgrade, Studentski Trg 12/V, 11158 Belgrade, Serbia; 7 Fuel and Combustion Laboratory, Faculty of Mechanical Engineering, University of Belgrade, Kraljice Marije 16, 11120 Belgrade, Serbia; nmanic@mas.bg.ac.rs

**Keywords:** devolatilization, kinetics modeling, autocatalysis, cellulose triacetate, carbonization, mesoporous charred carbon

## Abstract

Thermo-chemical conversion via the pyrolysis of cigarette butt (CB) filters was successfully valorized and upcycled in the pre-carbonization and carbonization stages. The pre-carbonization stage (devolatilization) of the precursor material (cellulose acetate filter, r-CAcF) was analyzed by micro-scale experiments under non-isothermal conditions using TG-DTG-DTA and DSC techniques. The results of a detailed kinetic study showed that the decomposition of r-CAcF takes place via complex mechanisms, including consecutive reaction steps and two single-step reactions. Consecutive stages include the *α*-transition referred to as a cellulose polymorphic transformation (cellulose I → II) through crystallization mechanism changes, where a more thermodynamically ordered system was obtained. It was found that the transformation rate of cellulose I → II (‘cellulose regeneration’) is strongly affected by the presence of alkali metals and the deacetylation process. Two single-step reactions showed significant overlapping behavior, which involves a nucleation-controlled scission mechanism (producing levoglucosan, gaseous products, and abundant radicals) and hydrolytic decomposition of cellulose by catalytic cleavage of glycosidic bonds with the presence of an acidic catalyst. A macro-scale experiment showed that the operating temperature and heating rate had the most notable effects on the total surface area of the manufactured carbon. A substantial degree of mesoporosity with a median pore radius of 3.1695 nm was identified. The presence of macroporosity on the carbon surface and acidic surface functional groups was observed.

## 1. Introduction

Since the 1950s, the production of plastics has surpassed the production of almost all other materials. Most of the plastic that is produced is designed to be thrown away after only one use. As a result, plastic packaging makes up approximately one-half of the world’s plastic waste. Most of this waste is generated in Asia, while the United States of America (USA), Japan, and the European Union (EU) are the world’s largest producers of waste plastic packaging per capita [1]. The ability to deal with plastic waste has already been mastered. Only 9% of the plastic waste the world has ever produced is recycled. Most end up in landfills or the environment. If current patterns of consumption and waste management practices continue, by 2050, there will be approximately 12 billion tons of plastic waste in landfills and in the environment. By that time, if the growth of plastic production continues at the current rate, the plastics industry may account for 20% of the world’s total oil consumption [1].

The most common single-use plastics found in the environment are cigarette butts, plastic drink bottles, plastic bottle caps, food wrappers, plastic grocery bags, straws, and stirrers, other types of plastic bags, and foam containers. Plastic waste has caused great damage to the environment on land and at sea. It is estimated that as many as 12.7 million tons of plastic end up in the ocean every year, where it threatens wildlife. As plastic accumulates and breaks down into smaller pieces in the ocean and seabed, plastic pollution enters the food chain. The huge consumption of plastics and their irregularity dumping is quickly turning our oceans into the world’s largest landfills, endangering marine life, and eventually entering the food chain as plastic breaks down in the oceans and turns into microplastics. In the Mediterranean, plastic remains were found in the stomachs of small fish, sea birds, turtles, and gastropods. Scientists estimate that there are 1.455 tons of plastic in the Mediterranean Sea and compare it to a so-called “ocean vortex” of plastic [2].

According to the announcement of Kurmus and Mohajerani [3], the industrial cigarette is made of various parts, where the most important part represents the filter. The filter is made of cellulose acetate (CAc) thin fibers, designed to increase filtering efficiency by stopping a portion of the smoke from being inhaled. Approximately 97% of cigarettes contain filters. CAc fibers have a diameter of approximately 20 μm, and they are treated with titanium dioxide, TiO_2_ (to reduce the luster of the fibers). Afterward, they are packed in 15,000-fiber units, with the use of glycerol triacetate (triacetin (TA)) as a binding agent, creating a filter (TA is commonly used as the plasticizer for cellulose acetate filters; to obtain a sufficient hardness target, values for TA vary typically, between approximately 6% and 9% of the total filter weight [4]). Many scientific reports [5,6,7] show that cellulose acetate is a different form of cellulose because of its plasticization. Cellulose is biodegradable by any organisms using the cellulase enzyme. Cellulose acetate (CAc), due to the modifications of cellulose, has a large degree of acetate substitution (more or less ~2.45), and this increases the filtering efficacy but strictly limits its biodegrading potential. Namely, the chemical action makes CAc inaccessible to microbial decomposition, and, thus, the current product is only photodegradable. Furthermore, CBs have a low nitrogen (N) content (having a carbon (C) to nitrogen (N) ratio C:N of approximately 200), and this further limit the possibility of microbial decomposition.

A special problem is the toxicity of CBs itself. A large number of highly harmful compounds are released into the environment by cigarette particulate matter (tar) and smoking, which come from an active smoking procedure. Among such substances, a great threat comes to us, because among them, there are more than fifty compounds that are cancerogenic to humans. Examples of the chemicals found are as follows: Nitrogen oxides, benzene, acetaldehyde, formaldehyde phenol, argon, polycyclic aromatic hydrocarbons, ammonia, carbon monoxide, pyridines, and acetone [8]. In addition to all the listed chemicals, there is also ash. Not all chemicals in the cigarette produced in the combustion process remain in the CB. There are a number of compounds that are retained in CB and then emitted into the air. On the other hand, some compounds are too volatile to be retained in the filter, while others are retained but are subsequently very unlikely to be emitted from CBs because they are not volatile enough. It should be noted that in CBs, very toxic metals are left behind, such as cadmium (Cd), manganese (Mn), copper (Cu), and zinc (Zn) up to concentrations of 104 μg·g^−1^ of CB, then mercury (Hg) and arsenic (As) with concentrations measured up to 0.051 mg·kg^−1^ and up to 0.651 mg·kg^−1^ of CB, respectively. Further, zinc (Zn) and iron (Fe) have been found in the cigarette tobacco, the filter, and the ash [9].

Among the scientific literature, there are new articles that were published on recycling CBs, and many of them have encouraging results. However, some of the approaches have shown promising results in terms of CBs and cigarette filters (CFs) recycling in the fired clay bricks [10] and asphalt concrete [11], as the active carbons (ACs), as the sound of absorbing material [12,13,14,15], the thermal insulator [16], anode materials manufacturing [17], supercapacitors production [18], the fabrication of the environmental energy-enhanced solar steam evaporator [19], etc. Additionally, CBs have found application in the synthesis of nanostructured MnO_*x*_ catalysts for NO_*x*_ reduction within flaming combustion and smoldering processes [20], as well as in the CB-assisted combustion synthesis of modified CaO -based sorbents for high-temperature CO_2_ capture [21]. Considering such wide use of CBs and/or CF for the purpose of obtaining more valuable products through recycling, a special place was occupied by the char (carbonaceous) (or “bio-char”) and activated carbons (ACs) (physically or chemically modified surfaces of chars (bio-chars)) materials production with a porous structure. The precursor for AC fabrication represents the carbonaceous porous structures called chars, which were usually obtained by thermo-chemical conversion in the absence of oxygen at elevated temperatures, i.e., by the pyrolytic process (pyrolysis) [22]. Pyrolysis represents one of the most effective ways to recycle plastic waste into value-added carbon products (char or bio-char), especially those that contain cellulose derivatives such as filter parts of CBs. With this approach, the contribution to understanding bio-char’s usage in the remediation of hazardous pollutants and/or for energy purposes can be considered. Pyrolysis is a very versatile process that is used in many applications, directly or indirectly. Unfortunately, this versatility is also responsible for the fact that this process is often studied only in the context of one specific research field [23]. Pyrolysis has found wide application in four main areas, such as carbon material production, fabrication of carbon-micron nanodevices, chemical analysis, and waste treatment. In our work, the focus is on the interconnection of the first and fourth areas, but as alternative options, depending on the quality of the measurement data and the obtained results. Namely, in the given work shown here, the center of gravity was turned towards the physical aspects of the considered process. In terms of physical changes (e.g., phase, density, and morphology), heavy hydrocarbons adopt one of two possible mechanisms, known as coking or charring, during their pyrolysis process; the stated principles are explained in detail elsewhere [24].

The main goal of this research is to enable the upcycling of used cigarette butt filters (CBFs) into economically valuable highly porous carbon (HPC) production with a variety of applications using the pre-carbonization and carbonization stages in a multi-scale experimental study. The pre-carbonization stage was analyzed using thermal analysis techniques such as thermogravimetry (TG), derivative thermogravimetry (DTG), differential thermal analysis (DTA), and differential scanning calorimetry (DSC), with simultaneous measuring at three different heating rates (*β* = 10, 20, and 30 K/min) and covering the range of *β*’s, which belong to the slow pyrolysis regime. As test samples, mechanically separated and shredded CAc fibers from CBs were used for thermal measurements. Additionally, a detailed description of the thermal properties of the initial precursor during its transformation was presented. Further, no aqueous solutions or chemicals were used to separate the filter from other components of CBs (no pre-swelling with alkaline solutions was used). In the current study, during this trial, no chemical leaching was used to remove metal contaminants, all in order to examine the influence of the present metals and other ingredients on the physicochemical properties of the obtained carbon. The characteristics of the CAc-precursor and the obtained product were investigated by structural analysis via X-ray diffraction analysis (XRD) and spectroscopy analysis using the FTIR (Fourier-transform infrared) technique. The instrumental analyses were performed after the end of the carbonization stage when the desired product was synthesized and implemented at a large scale in a horizontal tube carbonization reactor. Also, for the observation of microstructural changes during the carbonization stage, scanning electron microscopy—energy dispersive X-ray spectroscopy (SEM-EDX) was applied. The porosity investigation for the manufactured carbon material was performed using the Brunauer-Emmett-Teller (BET) method, encompassing specific surface and pore size distribution analyses.

From the theoretical aspect, which is related to the evaluation of the thermal decomposition kinetics of the CAc-precursor, the isoconversional (model-free) approach [25] was applied. This kind of analysis is significant in understanding the decomposition behavior of such materials and their thermal decomposition kinetics, which are crucial for processing and further application. Kinetic parameters (the activation energy (*E_a_*) and logarithm of the pre-exponential (frequency) factor (*logA*)), including the pyrolysis process optimization, were determined by the application of Friedman’s (FR) model-free (isoconversional) method and advanced Vyazovkin’s model-free (isoconversional) method. The actual evaluation is performed using thermo-analytical data at the various heating rates under non-isothermal (dynamic) conditions. Furthermore, in order to determine the precise reaction mechanism of the pyrolysis process, the model-based (model-fitting) approach was applied, which enables a comprehensive analysis of chemical reactions that take place in the reaction system. This approach gives the possibility of obtaining detailed kinetic information about complex heterogeneous processes, that may consist of individual reaction steps, where each step can be individually connected to another reaction step in a consecutive, competitive, and/or independent manner within the process scheme.

This research provides insight into the efficient valorization of CBs filters based on cellulose derivate precursors through the one-step carbonization of harmful plastic waste into solid carbon material with high porosity, offering in that way the possibility to make a comparison between various samples manufactured so far for their practical application.

## 2. Materials and Methods

### 2.1. Raw Material and Sample Preparation

**Figure 1:**
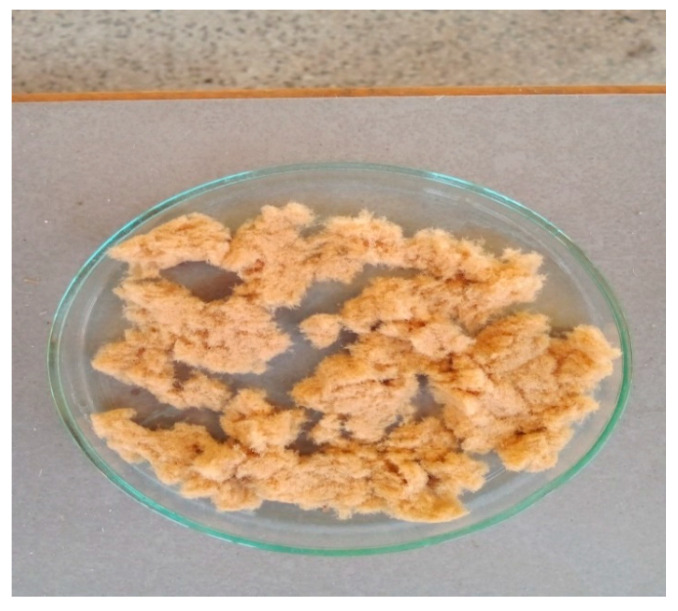
CAc-fibers of the used CB filters which were utilized in the experimental study.

The collected cigarette butts (CBs) from the same manufacturer (the same cigarette brand from the USA Company) were used as raw testing material. CBs were cleaned on the cardboard and covered it. Subsequently, the cigarette filters (CFs) (which are previously unwrapped from the paper) that are primarily cellulose acetate (CAc) fibers are mechanically separated from the rest of the CBs (note: separation of filters from the ash, the remaining tobacco, and the paper was performed by hand) (all CFs produced in the USA are made up of cellulose acetate (CAc) fibers containing delustrant, titanium dioxide (TiO_2_), as well as triacetin (TA) (glycerol triacetate), which is added to the fiber as the fiber binder (plasticizer)). The unwrapped filters were used for further operations. Thereafter, fibrils were chopped with scissors into smaller pieces and then put in a mill, where they were further ground. Figure 1 shows cellulose acetate (CAc) fibers out of a bundle of CB filters that were used in the experimental analyses.

### 2.2. The Pre-Carbonization Process Analysis Monitored by Simultaneous TG-DTG-DTA Measurements and Thermal Stability Tests

The pre-carbonization tests were performed by simultaneous non-isothermal thermogravimetry (TG), derivative thermogravimetry (DTG), and differential thermal analysis (DTA) using a SETARAM SETSYS Evolution 1750 instrument (7 Rue de l’Oratoire, Caluire-et-Cuire, France). The high-purity argon (Ar) gas (purity of 99.999%; SETARAM SETSYS) was used as a protective atmosphere. The samples with a mass of approximately 6 mg were heated at three different heating rates, *β* = 10 K/min, 20 K/min, and 30 K/min, in an argon (Ar) atmosphere (with a gas flow rate of *φ* = 20 mL min^−1^), in a temperature range from 30 °C up to 700 °C. Argon gas was chosen because, when it uses a wide range of temperatures, nitrogen can be less efficient than Ar as a result of the narrower range of optimal heating rates used (also, there is a detection system that prefers Ar because of the detection principle). To minimize air contact with the sample during its inserting, the oven environment of the thermogravimetric analyzer and the oven interior were permanently flushed with Ar (~80 mL min^−1^). The duplicate non-isothermal runs were performed under similar conditions, and the data were found to overlap with each other (including the control measurement for each heating rate used, with approximately the same sample mass), indicating satisfactory reproducibility. The calibration procedure was performed according to the manufacturer’s instructions and measurements with empty corundum crucibles in order to correct the measured signals and handle the mass balance deviations.

In addition to thermal characterization of a given material, TA Q1000 DSC Tzero™ (Thermal Analysis Instruments—TA Instruments LTD, 159 Lukens Drive, New Castle, DE, USA) equipment (which operates in the temperature range of Δ*T* = −180–725 °C with a sensitivity of 0.2 μW, where calibration is carried out via the instrument control software) was used for the determination of glass transition temperature (*T_g_*). The DSC test was performed with a sample mass of 3.30 mg at a heating rate of *β* = 10 K/min and under an argon (Ar) atmosphere (the purge and protective gas were *φ* = 20 mL min^−1^) to avoid any oxidation reactions. The DSC measurement was conducted through the double scan mode from +21 °C to +377 °C, where the *T_g_* point was estimated using the mid-point curve method, within a detected sensitive DSC signal change.

#### 2.2.1. The Pre-Carbonization Performance Indices

For describing the releasing performance of volatiles quantitatively, the devolatilization index (*D_i_*) was calculated in accordance with the following formula [26]:

(1) $$D_{i}=\frac{R_{max}}{\left(T_{in}T_{max}\Delta T_{1/2}\right)},$$

where *R_max_* is the maximum decomposition rate (%/min), while *T_in_* and *T_max_* are the initial devolatilization temperature and the maximum mass loss temperature, respectively; Δ*T*_1/2_ represents the temperature interval when the value of *R*/*R_max_* was ½ (i.e., when the instantaneous mass loss rate equals half of the *R_max_*). The devolatilization index (*D_i_*) is determined by the highest mass loss rate, where the decomposition is the largest, and is regarded as a measure to evaluate the pyrolysis ability of the samples of interest. The higher *D_i_* value generally indicates an easier pre-carbonization process and better pyrolysis performance.

The second quantitative index related to the evaluation of the considered process represents a heat-resistance index (*T*_HRI_) [27]. The heat-resistance index was the value used to evaluate the thermal resistance of the polymer. The HRI index can be calculated using the following formula:

(2) $$T_{\mathrm{HRI}}=0.49\times \left[T_{5}+0.6\times \left(T_{30}-T_{5}\right)\right],$$

where *T*_5_ and *T*_30_ are defined as temperatures at 5% and 30% of mass losses, respectively, and these temperatures can be extracted from TG data. If the *T*_HRI_ value rises as *β* increases, this is an indication that the thermal resistance increases with a heating rate.

#### 2.2.2. Isoconversional Kinetic Analysis of Pre-Carbonization Process

The fundamental equation for any kinetic study in the condensed phase is usually described by Equation (3):

(3) $$\frac{d\alpha }{dt}=k\left(T\right)\cdot f\left(\alpha \right),$$

where d*α*/d*t* is the conversion rate of decomposition at a constant temperature, *k*(*T*) is the decomposition rate constant, and *f*(α) represents the reaction (kinetic) model. The degree of conversion (or the conversion) can be obtained from the mass ratios at a given temperature or time, expressed as α = (*m*_o_ − *m_T_*)/(*m_o_* − *m_f_*), where *m_o_* is the initial mass, *m_T_* is the mass obtained at the estimated temperature (or time), and *m_f_* is the final mass at the analyzed instant. To stipulate the value of the rate constant, *k*, it is necessary to apply the concept of the Arrhenius equation law, which calculates the variation of the rate constant of the chemical reaction with temperature. Using the equation *k*(*T*) = *A*·*exp*(−*E_a_*/*RT*), it is possible to demonstrate the relationship between kinetic parameters, i.e., activation energy and pre-exponential (frequency) frequency factor, where *A* [s^−1^ or min^−1^] is the pre-exponential factor, *E_a_* is the activation energy (J mol^−1^ or kJ mol^−1^), *R* is the universal gas constant (8.314 J·K^−1^·mol^−1^), and *T* is the absolute temperature (K). By combining the above equation with Equation (3), the conversion rate given by Equation (3) can be transformed into Equation (4):

(4) $$\frac{d\alpha }{dt}=Ae^{-\frac{E_{a}}{RT}}\cdot f\left(\alpha \right),$$

where under non-isothermal experimental conditions, the time dependence can be eliminated by introducing the heating rate *β* = d*T*/d*t*, so the above equation in dynamic conditions takes the form:

(5) $$\beta \cdot \left(\frac{d\alpha }{dT}\right)=Ae^{-\frac{E_{a}}{RT}}\cdot f\left(\alpha \right),$$

where *β*·(d*α*/d*T*) ≡ (d*α*/d*t*) is the non-isothermal (dynamic) decomposition rate.

Isoconversional methods are widely used to study the non-isothermal decomposition of solid materials, so through them, it is possible to estimate the activation energy (*E_a_*) values independently of other parameters that constitute the kinetic triplet [*E_a_*, *A*, *f*(*α*)]. Kinetics analysis is based on the isoconversional principle, including methods such as the differential Friedman’s (FR) isoconversional method [28] and the advanced Vyazovkin’s (VY) isoconversional method as a universal kinetic approach [29,30].

Friedman’s (FR) method is considered the most efficient, achieving more accurate results when compared to other differential methods. The values of *E_a_* can be used through adjustments in relation to *ln*(d*α*/d*t*) vs. 1/*T*, whose slope of the straight line will determine the value of the activation energy (*E_a_*). The calculation for this new rate is performed from the linear relationship of the curve generated between the data of the analysis of time (s) vs. the actual sample temperature. From the slope of the line, the real heating rate (the real *β*) can be found. Such a method must be applied, according to Equation (6):

(6) $$ln{\left(\frac{d\alpha }{dt}\right)}_{\alpha ,i}=ln\left[A_{\alpha }\cdot f\left(\alpha \right)\right]-\frac{E_{a,\alpha }}{T_{\alpha ,i}}$$

where “*i*” represents the *i*-th heating rate used, at a given constant conversion value, i.e., *α* = const.

The Vyazovkin’s (VY) isoconversional method is a widely recommended integral approach for the accurate determination of activation energies *E_a_* [30]. The VY method proposes an exact equation (non-linear) based on the general assumption, that the reaction (kinetic) model is independent of the heating rate. The activation energy at a specific conversion value, α, is obtained by determining the *E_a_* value that minimizes Equation (7):

(7) $$\sum_{i=1}^{n}\sum_{j\neq i}^{n}\frac{I\left(E_{a,\alpha },T_{\alpha ,i}\right)\cdot \beta_{j}}{I\left(E_{a,\alpha },T_{\alpha ,j}\right)\cdot \beta_{i}}=min$$

where

(8) $$I\left(E_{a,\alpha },T_{\alpha }\right)=\int_{0}^{T_{\alpha }}e^{-\frac{E_{a,\alpha }}{RT}}dT.$$

In the above equations, *E_a_*_,*α*_ and *T*_α_ are the activation energy and the temperature at a conversion α, respectively, obtained from independent experimental runs *i* and *j* and performed at different heating rates, *β*’s. The integral is numerically evaluated by using the trapezoidal rule and a uniform grid spacing that is continuously decreased until a difference in the integral values is smaller than 10^−6^, between the consecutive interactions. Different activation energy values are then used in the above equations, and the activation energy for the process is determined as the value of *E_a_*_,*α*_, which gives the lower result for Equation (7). For all calculations, the highly sophisticated software NETZSCH Kinetics Neo (product version: 2.6.0.1, 2022) was used. The software allows the kinetic study to be performed by using thermal analysis techniques, such as TG, DTG, and/or DSC. For established isoconversional methods, in order to estimate the pre-exponential factors as *logA* values (software has the ability to transform “*ln*” data into “*log*” scale data, where it normally operates), the actual software uses the kinetic compensation effect (KCE) model [31]. The reliability of the modeling using Kinetics Neo software relies on accurate and reproducible thermal analysis data, which enables the numerical optimization of the investigated process and represents the model-free method (being used FR results) using the non-linear least squares optimization. For a closer look at the mentioned procedure, readers are referred to ref. [32]. In both model-free (isoconversional) and numerical optimization methods, the reaction type is not assumed. All calculations were performed in accordance with recommendations for collecting experimental thermal analysis data for kinetic computations, published by the International Confederation for Thermal Analysis and Calorimetry (ICTAC) [33].

#### 2.2.3. Model-Based (Model-Fitting) Kinetic Analysis

TG experiments are very widespread due to their exceptional flexibility and build performance as well as their ability to provide important characteristic decomposition temperatures. Likewise, the thermal analysis techniques enable the application of kinetic analysis, which would lead to the accurate determination of the process mechanism scheme. In the case of multi-step heterogeneous processes, it is often difficult to suggest a physical and geometrical model that corresponds to the character of the process during its entire course in the temperature regime under study. In macromolecular systems (where molecules of different sizes can participate), kinetic modeling represents a challenging task, so, in that case, the physical and mathematical description of the overall process through the use of various kinetic models is recommended. The use of relationships between kinetic parameters enables the process to obtain the desired numerical characteristics. So, without an adequate analysis of the results, it is impossible to give them a strict physical meaning. In order to provide insight into the physical meaning of phenomena that take place during heterogeneous processes, it is necessary to have a developed kinetic tool that reliably clarifies given events. For this purpose, various kinetic software programs have been developed that can be used to set up the appropriate reaction scheme of the process that corresponds to the real physicochemical transformations of the system under investigation. One such software is Kinetics Neo, which implements model-based kinetic analysis, permits the estimation of kinetic parameters using cutting-edge mathematical calculations, searches for the most statistically probable type of function to describe the actual process, and performs predictive computations. This software also contains a peak separation program for decomposing multimodal curves into their separate components. Concerning the above-described kinetic approach, the theoretical background related to model-based calculations can be seen in Supplementary Materials (Section SI).

### 2.3. Carbonization Process

Carbonization parameters, such as temperature, heating rate, and residence time, have effects on the final, synthesized porous carbon. There is a growing interest in understanding the effects of carbonization parameters on the final BET surface area of the produced charred carbon. Most studies have used one factor at a time rather than changing all factors simultaneously. This approach has drawbacks, such as statistical analysis in terms of interactions between various factors and inefficiency in the accuracy level of the produced results, while often requiring more experiments. However, the experimental “design” largely depends on the type of raw material tested, and the possibility of achieving the most homogenous sample for experimental work is closely related to the feedstock particle size (closely related to thermal conductivity and the heat transfer effect). However, in most cases, it turned out that the carbonization temperature represented the most influential factor in producing a carbon porous structure. In the case of thermoplastic polymers, charring is a crucial process. Namely, the primary parameter, which concerns the reduction of heat release rate, represents the development of multi-layered carbonaceous structures on the surface of fibrillar material, thus switching the non-charring behavior of thermoplastic polymers to that of charring materials. In that case, the high temperature intensified action has a decisive role through the polymer melting at elevated temperature and the accumulation caused by polymer ablation. Therefore, in this study, the one-step high-temperature carbonization procedure was chosen for low heating rate ramping and moderate residence (reaction) time. This approach is novel for this type of feedstock, for the production of highly porous charred carbon (without activation, physically or chemically), but it should be considered an initial (preliminary) stage for further research in this direction.

#### Carbonization Experiment in the Horizontal Tube Furnace

The one-step carbonization process (on a lab-scale level) was employed to obtain the carbon material from cigarette filters, which are primarily composed of CAc. As previously described, the CBs are collected, and without any pretreatment, the coated papers of the butts are carefully removed to obtain filters. After these procedures (without any rinsing), carbonization was started. The carbonization process was carried out in a stainless steel fixed-bed reactor (Protherm Furnaces, model PTF 16/38/250, Ankara, Turkey). About 10 g of the initial material (Figure 1) was placed in a carbonization reactor. During the carbonization process, purified nitrogen (N_2_) at a flow rate of *φ* = 300 cm^3^ min^−1^ was used as the purge gas. However, before heating, the system was flushed with dry nitrogen for *t* = 30 min to remove all traces of oxygen. The reactor temperature was increased from room temperature (RT) up to the desired operating temperature of *T* = 800 °C. When the operating temperature reached the desired value, it was held for *t_res_* = 1 h (static conditions). At the end of carbonization, the gaseous flow of N_2_ in the reactor was maintained during cooling until room temperature. The heating rate was constant, and its value was *β* = 4 °C min^−1^. In the following text, the raw material is marked with r-CAcF (raw-cellulose acetate filter), while the carbonized sample is marked with CAc800(1h).

### 2.4. Characterization of Raw Material and Derived Carbon Material

In this section, there will be reported instrumental techniques (methods) that were implemented for the physicochemical characterization of the raw material and the charred carbon that was produced in the actual experimental campaign. This characterization includes the implementation of the Fourier-transform infrared (FTIR) spectroscopy analysis, the X-ray powder diffraction (XRPD) analysis, the scanning electron microscopy (SEM)—energy dispersive X-ray (EDX) spectroscopy, and the Brunauer-Emmett-Teller (BET) theory in order to calculate the specific area of fabricated carbon.

#### 2.4.1. FTIR Analysis

Fourier-transform infrared (FTIR) spectroscopy spectra of r-CAcF and CAc800(1h) samples were recorded in absorbance mode with a Nicolet™ iS™ 10 FT-IR spectrometer (ThermoFisher SCIENTIFIC), equipped with Smart iTR™ Attenuated Total Reflectance (ATR) sampling technique. Recordings were performed in the wavenumber range of 400–4000 cm^−1^, at 4 cm^−1^ resolution, and in 20 s scan mode.

#### 2.4.2. X-ray Diffraction (XRD) Analysis

The r-CAcF and CAc800(1h) samples (in powdered form) were characterized at room temperature by the X-ray powder diffraction (XRPD) using an Ultima IV Rigaku diffractometer, equipped with CuKα1,2 radiations, a generator voltage of 40.0 kV, and a generator current of 40.0 mA. The range of 5°–80° 2θ was used for all powdered samples in a continuous scan mode, with a scanning step size of 0.02° and a scan rate of 10° per minute, using the D/TeX Ultra high-speed detector. The glass sample carrier for sample preparation was used. The PDXL2 (Ver. 2.8.4.0) software was used to evaluate the phase composition and for identification analysis. All obtained powders were identified using the ICDD database [34] and the ICSD database (https://icsd.products.fiz-karlsruhe.de/, accessed on 6 March 2023). The software package Powder Cell [35] was used to identify the phases present in the investigated samples. The TCH pseudo-Voigt profile function was given the best fit for experimental X-ray data.

#### 2.4.3. SEM-EDX Analysis

Scanning electron microscopy (SEM) with energy dispersive X-ray (EDX) spectroscopy was employed for morphological and micro-elemental analysis of the carbonized sample (CAc800(1h)). Measurements were performed on the JEOL JSM 6610 LV (JEOL, Akishima, Tokyo, Japan) scanning electron microscope in conjunction with the EDX detector model X-Max Large Area Analytical Silicon Drift, connected with INCAEnergy 350 Microanalysis (detection of elements *Z* ≥ 5, detection limit: ~0.1 mas.%, resolution: 126 eV). The element used for optimization was nickel. Before SEM measurements, the carbonized sample was positioned on the adhesive tape, which is fixed to the specimen tabs. Micrographs were recorded with different magnifications, while EDX microchemical analyses were done at selected points and areas. In the case of plastic or bioplastic materials, these groups of materials require preparation as non-conductive materials or materials with poor electrical conductivity, so, in that context, gold (Au) represents the material that is often used for the preparation of specimen measurements because of its high electrical conductivity and fine grain size, resulting in the corresponding high-resolution (h-R) images. Therefore, an (Au) coating was used in the experiments.

#### 2.4.4. Nitrogen Adsorption-Desorption Experiments and BET Analysis

The specific surface and pore size distribution was studied by the low-temperature nitrogen (N_2_) adsorption-desorption isotherm tests according to the Brunauer-Emmett-Teller (BET) method using a surfer gas adsorption porosimeter (Thermo Scientific Surfer Gas Adsorption Porosimeter, Thermo Fisher Scientific, Waltham, MA, USA). This is a very commonly used method to measure the specific surface area of the produced carbons, as in our case. The major assumption of BET theory was that adsorption takes place as a monolayer of adsorbed molecules on the surface of the adsorbent (furthermore, other theories were developed, like in the case of physisorption of molecules with a weak interaction with the adsorbent, because it is necessary to take into account the formation of a multilayer of considered adsorbed molecules). To perform adsorption and desorption experiments, an inert gas, nitrogen, was used. The inert gas was taken from a nitrogen bottle and, thanks to a pump, pumped into the device. Before passing the sample into the sorption device, the carbonized sample was degassed, at the stations provided for this operation.

The specific surface area, *S*-BET, pore size distribution, d*V*(*r*), mesopores, including external surface area, *S_meso_*, and the specific micropore volume, *V_mic_*, for the carbonized sample were determined from the full recorded adsorption/desorption isotherms. Pore size distribution d*V*(r) was calculated from the desorption isotherm branch by applying the Barrett–Joyner–Halenda (BJH) model [36], while a mesopore surface and micropore volume were estimated using the *t*-plot method [37,38].

## 3. Results and Discussion

### 3.1. FTIR Results of Precursor (r-CAcF) and Carbonized Sample (CAc800(1h))

**Figure 2:**
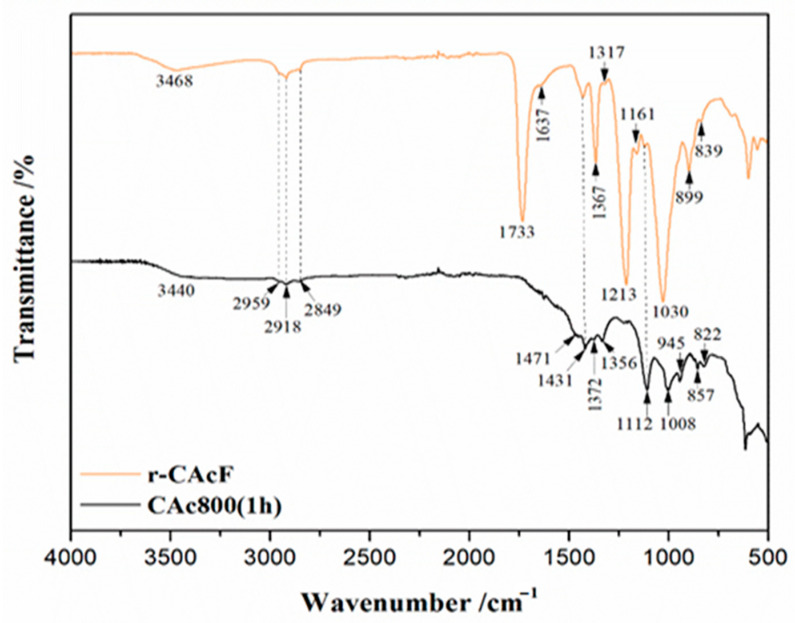
ATR-FTIR spectra of r-CAcF and CAc800(1h) samples.

The ATR-FTIR spectroscopy was applied to the raw (precursor) material, r-CAcF, and the derived carbon from the carbonization process, CAc800(1h), and their spectra are shown in Figure 2. In all presented FTIR spectra, the appropriate designation of characteristic vibrational bands and wavenumber positions is clearly highlighted.

The peak positions observed in the FTIR spectrum of r-CAcF indicate the structure of cellulose acetate (CAc). The peak at 3468 cm^−1^ is attributed to the ν(O-H) stretching vibrations of hydroxyl groups in CAc. Namely, the range of wavenumbers between 3500 cm^−1^ and 3400 cm^−1^ can be attributed to the OH stretching present on the cellulose surface [39]. It can be noted that the mentioned region is important because OH groups may give information about the formation of a hydrogen-bond matrix inside the actual structure since OH groups can form hydrogen bonds with not only C=O (see later) but also with other groups, as, for example, another OH group or –O– group [40]. So, the existence of the FTIR band at 3468 cm^−1^ in the r-CAcF sample (Figure 2) can be related to intramolecular hydrogen bonds [41]. The region between 2959–2849 cm^−1^ corresponds to ν(C-H) (a)symmetric stretching vibrations of methyl and methylene groups in polymer chains of cellulose, while the δ(C-H) bending vibrations for those groups were observed at 1431 cm^−1^, 1367 cm^−1^, and 1317 cm^−1^. Also, the band at 1367 cm^−1^ corresponds to the bending vibration characteristic for the methyl in the acetyl groups [42]. Further, the FTIR strong intensity signals at 1733 cm^−1^, are characteristic of ν(C=O) stretching vibrations of ester groups [39,43]. The weak peak at 1637 cm^−1^ indicates the δ(O-H) bending vibrations of OH and absorbed water by the cellulose precursor. In addition, high-intensity peaks at 1213 cm^−1^ were ascribed to the stretching vibrations of the acetyl ester group [44], while the broad strong band at 1030 cm^−1^, and weak signals at 1161 cm^−1^ and 1120 cm^−1^, appeared to be ν(C-O) asymmetric/symmetric stretching vibrations of ether groups [45]. The intensity at wavenumber positions of 899 cm^−1^ and 839 cm^−1^ were characteristics of C-O-H out-of-plane banding and *β*-glucosidic bonds, respectively. In addition, the vibrational band located at 603 cm^−1^, for r-CAcF (in Figure 2, this band is not marked with an arrow in the far-infrared region) belongs to the -C=O stretching vibration in the triacetin (plasticizer) molecule [46].

The characteristic groups for the carbonized sample (CAc800(1h)) result in FTIR signals with changes in shape, positions, and lower intensity (Figure 2). In the corresponding spectrum, due to the dehydration (removal of moisture) process, the intensity in the range of broad-band vibration of hydroxyl functional groups decreased (also dependent on the degree of carbonization). Simultaneously, the peak for the ester group disappeared, while the intensity of C-H and C-O vibrations decreased and shifted, indicating the cleavage of the glycosidic ring and the acetyl bond during the carbonation reaction [47,48]. Additionally, new vibration peaks at positions of 945 cm^−1^, 857 cm^−1^, and 822 cm^−1^ appeared (Figure 2), pointing to the formation of unsaturated bonds and aromatic structures [49]. Additionally, a noticeable peak at about 600 cm^−1^ (it is not designated in the CAc800(1h) FTIR spectrum (Figure 2)) can be attributed to the C-OH out-of-plane bending mode of aromatic compounds [50].

### 3.2. XRD Results

**Figure 3:**
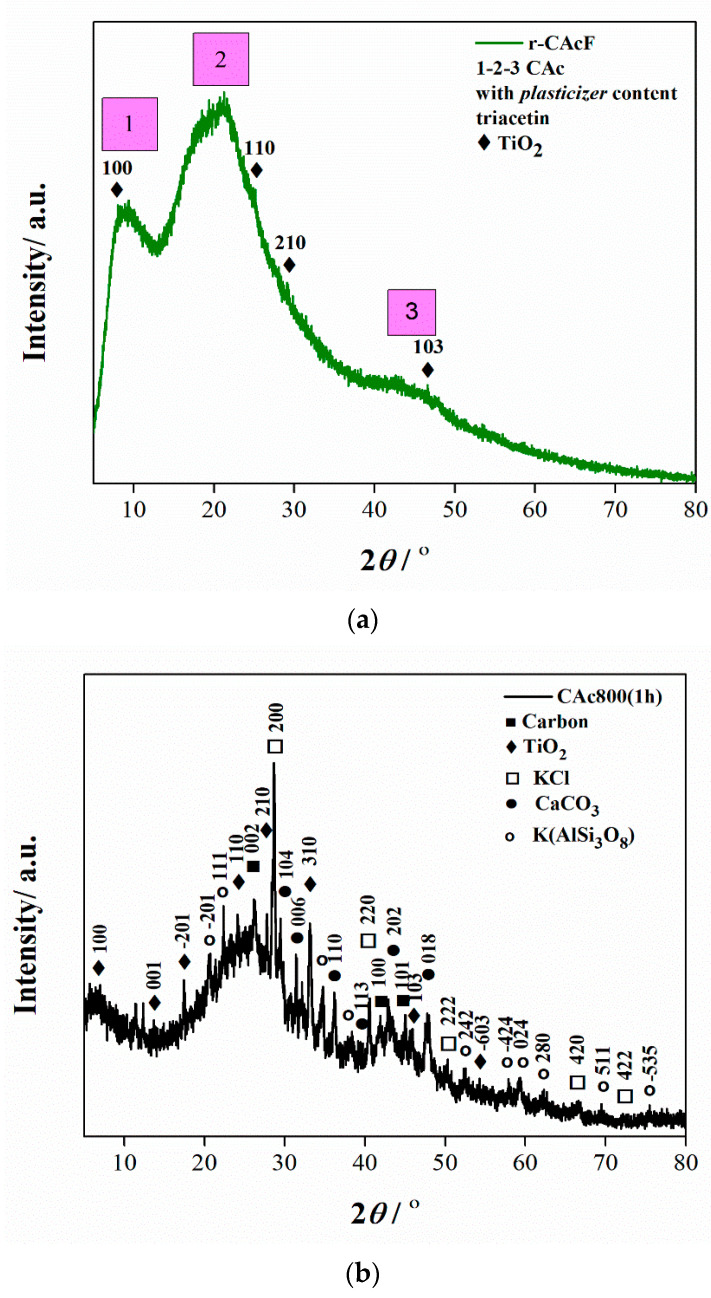
The XRD diffraction patterns of (**a**) r-CAcF sample, and (**b**) CAc800(1h) sample.

The X-ray diffraction (XRD) pattern of the r-CAcF sample is shown in Figure 3a. Two phases are observed in the form of cellulose acetate (CAc), a plasticizer (triacetin) (TA), and titanium dioxide, TiO_2_. Namely, this diffractogram primarily reflects the structural image of cellulose acetate (CAc), which can be compared with the PDF card–00-062-1713, from the ICDD database. Although CAc, as a cellulose derivative, is the most abundant organic polymer on Earth, it has its own weaknesses. This is why it needs to be plasticized because its melting temperature (*T_m_*) is too close to its decomposition temperature [51].

An ideal plasticizer to mix with CAc is triacetin (TA), which was used to improve certain performance characteristics and/or reduce costs [52]. However, in the actual case, the CAc has been plasticized with TA, which suits PDF card–00-061-1409, but here (in the actual case considered), it cannot be determined in what proportion. That is why we do not have pronounced XRD peaks in Figure 3a, but three humps that include three regions: first at 2*θ* ≈ 10°, second at 2*θ* ≈ 20° and third at 2*θ* ≈ 40°, which indicate the short-range intermolecular order of the amorphous phase [51]. The absence of the pure crystalline phase in CAc occurs as a result of its plasticization with triacetin (TA). However, the existence of a peak at about 2*θ* ≈ 8.880° (it is not strictly marked in Figure 3a is characteristic of the semi-crystallinity (this peak may indicate a disorder when the cellulose is acetylated). With acetylation, acetyl groups may cause an increase in the inter-fibrillar distance and a break in the micro-fibrillar structure. The position of this peak suggests disorder generation, which confirms that the cellulose is acetylated [53]. Results feature a wide halo, about 2*θ* ≈ 20°, known as van der Waals or the amorphous region, which is characteristic of all organic polymers. By increasing the plasticizer content, the intermolecular hydrogen bond of CAc is broken and then interacts with CAc. This is probably the reason why instead of pronounced peaks in Figure 3a, we can see amorphous humps, which are the most affected by the effect of plasticization. Furthermore, intermolecular forces between polymer chains, space occupied by the main chain and side chains the movement of side chains, and density should not be neglected [54]. In this respect, the plasticizer dramatically changes the hydrogen bonding of the polymer network and affects its corresponding hump (Figure 3a). The few peaks with mildly expressed intensities that are positioned at around 2*θ* = 7.587°, 24.979°, 28.334°, and 46.525° pertain to the (100), (110) (210) and (103) planes, respectively (Figure 3a), belonging to the TiO_2_ (titanium dioxide; ICSD No. 657748), whose presence is expected in the considered r-CacF sample.

The X-ray pattern of the carbonized sample (CAc800(1h)) indicates the multiphase composition, and this is shown in Figure 3b. All narrow peaks with high intensities belong to TiO_2_, KCl (potassium chloride) (ICSD No. 154214), CaCO_3_ (calcium carbonate) (ICSD No. 158258), and K(AlSi_3_O_8_) (orthoclase–aluminum potassium trisilicate) (ICSD No. 90141), while the broader, weaker diffraction peaks indicate on the presence of graphite-type carbon (ICSD No. 617290). These wide-ranging peaks indicate a small grain size and incomplete grain shape and also suggest a weak crystalline structure [55]. Considering that the crystal structure was initially disturbed by the participation of the plasticizer, the weak crystal structure was formed as a consequence of the carbonization intensification. All present planes that appear as a result of the carbonization of the r-CAcF sample and that are connected with the formation of carbon (Figure 3b) are characteristic of the carbonized CFs [56].

### 3.3. Thermal Stability of r-CAcF and Kinetic Analysis of Pre-Carbonization Stage (Devolatilization/Pyrolysis Kinetics Modeling)—Micro-Scale Experiments

#### 3.3.1. TG-DTG-DTA Results

**Figure 4:**
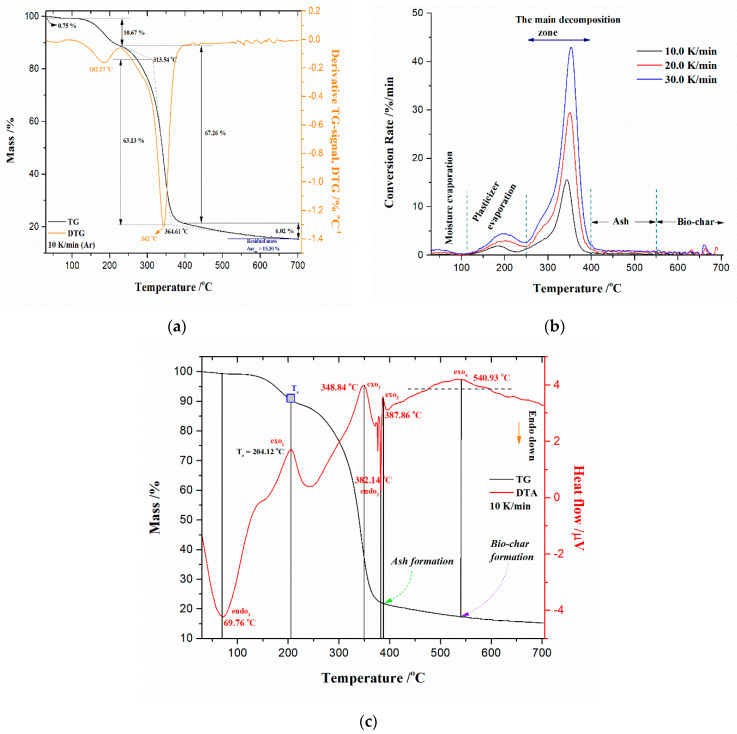
Thermal properties of r-CAcF sample monitored in argon (Ar) atmosphere: (**a**) Simultaneous TG-DTG curves at *β* = 10 K/min, with marked characteristic temperatures and sample mass losses at given process stages (the residual mass was also designated), (**b**) Conversion rate (absolute) curves (%/min) at various heating rates (*β* = 10, 20, and 30 K/min) with marked reaction phases during thermo-chemical conversion, and (**c**) Simultaneous TG-DTA curves at 10 K/min, with marked thermal effects during the thermo-chemical conversion process (the reaction point-check temperatures were also designated).

The thermal analysis measurements were performed in order to define the changes during the thermal decomposition of the precursor based on CAc (r-CAcF). The simultaneous TG-DTG curves for the devolatilization process of an r-CAcF sample at 10 K/min in an argon (Ar) atmosphere are plotted in Figure 4a (TG-curves at different heating rates (*β* = 10, 20, and 30 K/min) with designation of the main process stages and corresponding residual mass values (Δ*m_res_*) are shown in the Figure S1 (Supplementary Materials)). It can be seen from Figure 4a that there is a small sample mass loss of approx. ~0.75% with a slight inclination on the DTG curve below 100 °C, which can be attributed to moisture evaporation (desorption) accumulated on the surface of the sample. After that, there is a significant mass loss (~10.67 °C) manifesting as a DTG peak at 182.27 °C (Figure 4a), and this reaction stage is attributed to the thermal decomposition of the plasticizer [51]. In the third reaction stage, there is the largest mass loss of the sample at ~63.13%, which represents the main decomposition stage. This stage is characterized by a DTG peak located at a temperature of 342 °C, which is attributed to the thermal decomposition (pyrolysis) of cellulose acetate (CAc) [57]. Namely, it can be assumed that the initial decomposition temperature of CAc is 313.54 °C and the maximum mass loss rate temperature is 364.61 °C, where the mass loss between them amounts to ~67.26% (Figure 4a). In the fourth reaction stage, the sample mass loss is ~6.02%, with a residual mass of ~15.30% at 700 °C (Figure 4a). The actual stage is characterized by processes of ash formation (generation of a complex mixture of heavy metals, which is retained after the degradation of the basic material, enabling metal recovery—it should be emphasized that it is not the ash created by the tobacco combustion process (it was removed from the CB filter previously)) and formation of carbon residue (bio-char). All stated thermo-chemical conversion stages of the investigated material are clearly marked on corresponding conversion rate (absolute) curves as a function of process temperature at various heating rates, which are shown in Figure 4b.

Based on the position of peaks appearing on the conversion rate curves (Figure 4b), it seems that the pyrolysis step related to plasticizer thermal decomposition showed a certain dependence on the heating rate. On the other hand, the conversion peaks located inside the main decomposition zone showed moderate dependence on the heating rate. The maximum temperature peak shifts are in proper order with an increasing heating rate (where the reaction rate also increases) in the *T*-range between 300 °C and 400 °C, where deacetylation reactions may occur [58]. The charring process took place at higher temperatures, above approx. 550 °C, and was highly dependent on CAc degradation reactions. The residual mass values (Δ*m_res_*) in the last stage showed less variation with changing the heating rate (Figure S1). By increasing the temperature above 600 °C until the very end of the process, the carbon yield amounts to about 10–15% (Figure S1). Finally, the last one, where there was a slow mass loss by increasing the temperature up to 700 °C (Figure 4a,b), belongs to the cracking reaction of C–C bonds, which is a slower step.

The comparative analysis of physicochemical phenomena derived from TG (thermogravimetry) and DTA (differential thermal analysis) recordings closely related to r-CAcF thermal decomposition can give more detailed information about the thermal effects that then occur. This is important, considering the thermal properties of both the plasticizer and CAc. The appropriate simultaneous TG-DTA curves for the pyrolysis (devolatilization) process of the r-CAcF sample at *β* = 10 K/min are shown in Figure 4c. From the presented DTA scan, it appears that there were both exothermic and endothermic effects during the decomposition of the r-CAcF. Namely, the broader endothermic peak (endo_1_) located at 69.76 °C (Figure 4c) is attributed to the water (H_2_O) desorption from the polysaccharide structure. With an increase in the temperature, at a position of *T* ~204.12 °C, the exothermic event (exo_1_) appeared (Figure 4c), and this can be attributed to CAc crystallization (marked as *T_c_* in Figure 4c) [59,60]. This event suggests that part of the material can be crystallized under appropriate conditions, and it is a clear indication of the change in the degree of substitution (DS) [60]. This term is usually used in cellulose chemistry, where each anhydroglucose (β-glucopyranose) unit has three reactive (hydroxyl) groups. The DS can therefore range from zero (cellulose itself) to three (fully substituted cellulose). In the case of CAc, the DS is the average number of acetyl groups attached per anhydroglucose unit. In total perspective, the eight possible anhydroglucose units (AGU) of CAc may occur. So, for our investigated filter (r-CAcF), we are dealing with a typical cellulose derivative where the substitution of hydroxyl groups of cellulose are substituted by other functional groups (by the acetate). An accurate determination of DS for our sample will be presented in the next subsection related to the estimation of the glass transition temperature (*T_g_*). It should be pointed out that the observed exothermic events take place in the temperature range where plasticizer evaporation occurs. Namely, the plasticizer is incorporated into the amorphous parts of polymers, while the structure and size of any crystalline part remain unaffected [51]. The plasticizer is expected to lower the *T_g_* and reduce the modulus, tensile strength, hardness, density, etc. It is a low molecular weight (low-MW) chemical that specifically interacts with polymers and spreads them apart in order to increase the free volume in one system. The added plasticizer reduces polymer–polymer bonding and can provide more molecular mobility for the macromolecules [51].

By further increasing the temperature, it is observed a second, broad exothermic event (exo_2_), placed at the temperature point of ~348.84 °C (Figure 4c), which may be an indication of the secondary crystallization arrangement and also of the recombination of the hydrogen bonding [60]. This confirms observations identified from XRD analysis (Figure 3a) about the existence of intermolecular hydrogen bonding. The shape of this exothermic peak is dependent on the extent of the modification of the CAc structure. In any case, it must be pointed out that the acetyl groups of triacetin (TA) are supposed to interact with CAc via dipolar interactions and hydrogen bondings. The influence of these polar interactions could affect the *T_g_* value. Considering the FTIR spectrum in Figure 2 for the r-CAcF sample, based on the intensity of the FTIR band at 3468 cm^−1^, this band can be assigned to OH groups with weak hydrogen bonds. On the other hand, the weak H-bonded OH groups probably appear in concert, mainly with structural changes in the intrachain hydrogen bonds [61]. The H-bonding energies can play a significant role in observable molecular rearrangements, which can be discussed through the apparent reaction mechanisms within the kinetic analysis.

At the temperature of 382.14 °C, a sharp and narrow DTA peak appeared, indicating the endothermic event marked as endo_2_ in Figure 4c. This transformation indicates the melting of the acetylated sample [59]. At this point, we come to an important crossroads, where it is obvious that the material’s melting process overlaps with its decomposition process. Reactions that indicate these phenomena take place in a narrower temperature range, but it largely depends on the DS. According to these results, the realization of the competitive fusion phenomenon of crystallized material and the decomposition of cellulose derivatives may occur.

At the temperature position of approx. ~387.86 °C, immediately after the previously discussed transformation, there appeared to be an exothermic event (exo_3_), which can be attributed to the ash formation (Figure 4c). Within the framework of this reaction phase, the appropriate amount of metals and inorganic fractions remaining in the used CFs accumulates in the form of ash and can be called metal “contaminants”. There is an accumulation of alkali and alkaline earth metals in the form of carbonates and chlorides, as well as the existence of thermally stable TiO_2_, whose presence was identified in the carbonized sample by means of the XRD analysis (Figure 3b). The last phase was characterized by a broader exothermic effect, located at approx. ~540.93 °C (as exo_4_, Figure 4c), where the bio-char is formed. The presence of indicated metals and especially TiO_2_,, can have catalytic effects on the fabrication of the final solid product, acting on the morphology and surface properties of the synthesized carbon via carbonization.

#### 3.3.2. Determination of the Glass Transition Temperature (*T_g_*) by the DSC Testing Probe

The DSC test of the r-CAcF sample was used in order to determine the glass transition temperature (*T_g_*) through the mid-point curve method within the sensitive DSC signal changes described in Section 2.2. Figure S2 (Supplementary Materials) shows the DSC trace of the r-CAcF sample at a heating rate of 10 K/min, measured in the temperature range of Δ*T* = 21–377 °C. From the recorded DSC curve, the melting temperature (*T_m_*) was determined through the cross-section tangents approach. For the investigated sample, a *T_m_* value of 258.50 °C was obtained. The estimated *T_m_* value lies a little outside of the melting temperature range for commercial CAc with an acetylation content of approximately 40% and plasticized with glycerol triacetate (~230–250 °C) [52]. More reliable retrieval of the *T_m_* value from DTA measurements is difficult to determine, bearing in mind the above-presented discussion. The *T_m_* and *T_g_* values are most often determined by the DSC technique, but this technique is not so precise in comparison with, for example, the DMA (Dynamic Mechanical Analysis) technique. However, from the presented DSC experimental testing, the visible *T_g_* point cannot be detected (Figure S2). As a consequence of this fact, CAc has a narrow temperature window between its *T_g_* value and its decomposition temperature value. The temperature position where *T_g_* will appear depending on the type and concentration of plasticizer. Applying the procedure described in Section 2.2, the enlarged portion of the DSC trace where sustained sensitive DSC-signal changes are detected is shown in Figure S3 (Supplementary Materials). The procedure for determining *T_g_* according to the described method is shown schematically in the same figure. Based on the current investigation, the *T_g_* is determined to be *T_g_* = 182.68 °C (Figure S3). The estimated value of *T_g_* corresponds to CAc with a degree of substitution (DS) equal to 2.8 (DS = 2.8), so it can be supposed that we have cellulose triacetate (CTA). This result can be confirmed by the results previously presented in the XRD analysis (see above). The *T_g_* is important, considering the interpretation of the term miscibility, between the components (CTA and TA). The sharp *T_g_* transition in the single-point was not observed in our case, which is characteristic for miscible blends, but related to *T_g_*’s of both components. In the actual case, there is a broadening of the *T_g_* transition, which is characteristic of the heterogeneous dispersion of the components in the blend. Otherwise, partial miscibility (a limited character) may be excluded in this phase of the investigation. It should be mentioned that *T_g_* of DS = 2.5 exhibits a value of 190 °C [51], while in our considered case, the *T_g_* value amounts to 182.68 °C, so it is lowered to 7.32 °C, increasing the degree of substitution up to 2.8. In addition, the reduction in *T_g_* value creates a need for a larger amount of plasticizer, which would be incorporated into the CAc formulation. It was shown earlier that plasticization of CAc results in an increase in mass loss between 50% and 90%, where the low molecular weight plasticizers can be more effective, and that a right balance between hydrophilicity and plasticization efficiency (reduction in *T_g_*) is needed to increase the degradation rate of the CAc [62]. It should be emphasized that during thermal degradation, the plasticizer may diffuse through the polymer matrix, which can be affected by the progressive loss of the plasticizer that occurs during the degradation evolution [63].

#### 3.3.3. Thermal Stability and Devolatilization Indexes

Table 1 lists the values of the *D_i_* index and heat-resistance index (*T*_HRI_) at different heating rates (*β* = 10, 20, and 30 K/min) considering the pre-carbonization stage of the r-CAcF sample, developed on lab-scale measurements. The appropriate quantities attached to *D_i_* refer to the main decomposition zone designated on the absolute conversion rate curves and shown in Figure 4b. On the other hand, the appropriate quantities attached to *T*_HRI_ are derived from TG curves (Figure S1 (Supplementary Materials)).

From the results presented in Table 1, it can be observed that with an increasing heating rate, both quantities, *T_in_* and Δ*T*_1/2_, moved to higher values, elevating them to a higher temperature region. With an increase in the heating rate, the *D_i_* value became higher, and thus, the volatile release was much easier. Therefore, the *D_i_* value increases with an increase in the heating rate, indicating that the higher heating rate promotes the release of volatiles (thereby reducing the solid product). This should be expected in the temperature range where the endothermic peak endo_2_ occurs (Figure 4c) since that devolatilization reaction is an endothermic process while the formation of bio-char is an exothermic process (Figure 4c).

Namely, the higher heating rate decreases the residence time of the sample at lower temperatures and increases residence time at higher temperatures, correspondingly. Therefore, from the perspective of the reaction equilibrium, an increase in the heating rate is conducive to inhibiting the condensation reaction and promoting the devolatilization reaction. Additionally, with an increase in the heating rate, the maximum decomposition rate, *R_max_*, is accelerated and shifts to higher temperatures (Table 1, Figure 4b), mainly caused by the thermal hysteresis effect; the system is more easily decomposed at higher temperatures, so the values of *R_max_* and *D_i_* increase (Table 1). Additionally, the thermal stability of the r-CAcF sample was determined by TG through the determination of *T*_5_, *T*_30_, as well *T*_HRI_ quantities, respectively. Both values, *T*_5_ and *T*_30_, raise with an elevating heating rate elevating, but these values strongly depend on the degree of substitution (DS). Namely, the decomposition temperatures, *T*_5_ (Table 1), are smaller than those associated with the precursor sample with lower DS values [64]. Namely, it was found that the onset decomposition temperature for the cellulose triacetate sample with DS = 2.92 amounts to 252 °C [65], so we can see that *T_in_* values listed in Table 1 are very close to the indicated ones, and consequently, these values are highly appreciated for our sample with DS ~2.8. This is closely related to the crystallinity index. Hence, with an increase in DS, the crystallinity decreases [66]. In that sense, the decrease of crystallinity for our sample (Figure 3a) (the semi-crystallinity behavior) indicates the reduction of hydroxyl groups (which is indicated by the reduced intensity of the FTIR band located at ~3468 cm^−1^, and marked as ‘weak hydrogen bonds (Figure 2)), leading to less organization of the chains, decreasing intermolecular interactions through the hydrogen bonding, and thus decreasing the crystallinity. All this was confirmed by earlier results, using FTIR and XRD analyses. The mentioned facts are also reflected in the values of the heat-resistance index (*T*_HRI_) (Table 1). Based on the actual discussion, acetyl groups decrease the intensity of the formation of hydrogen bonds, and the lower temperatures (Table 1) are therefore needed for the beginning of the event of the glass transition temperature—*T_g_* (Section 3.3.2).

#### 3.3.4. Kinetic Analysis of Devolatilization Process

##### 3.3.4.1. Model-Free (Isoconversional) Results

**Figure 5:**
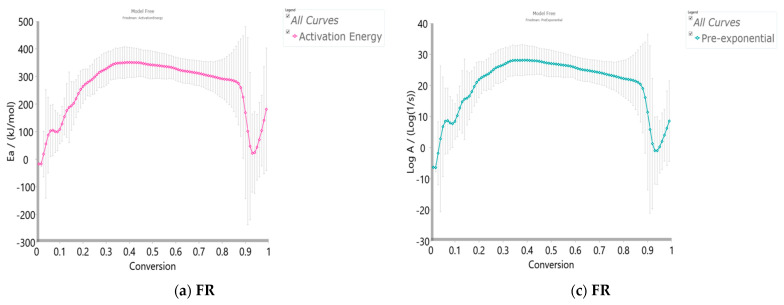

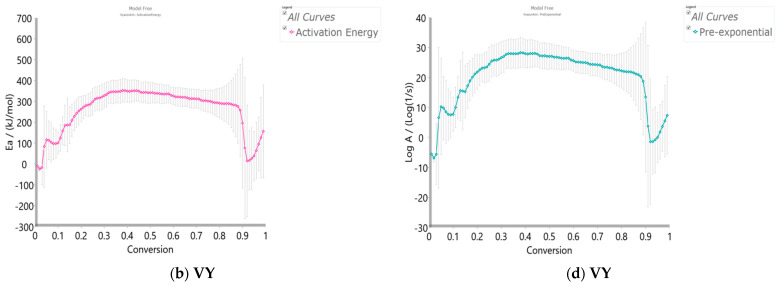
Isoconversional dependency of activation energies (*E_a_*) and the logarithm of the pre-exponential factors (*logA*) using Friedman’s (FR) (**a,c**), and Vyazovkin’s (VY) (**b,d**) model-free (isoconversional) methods, respectively, for the pyrolysis (devolatilization) process of the r-CAcF.

Figure 5a–d shows the isoconversional dependency of activation energies (*E_a_*) and the logarithm of pre-exponential factors (*logA*), respectively, for the pyrolysis process of the r-CAcF sample, obtained by the differential Friedman’s (FR) and Vyazovkin’s (VY) model-free (isoconversional) methods.

All isoconversional/model-free plots related to the behavior of *E_a_* and *logA* values (Figure 5a–d) during the pyrolysis show a very complex kinetic nature as the conversion progresses. It can be seen from the presented plots of both methods that there are several reaction regions where the kinetic parameters show the variation with a conversion (*α*). Such changes can be summarized by the following important items:

(a) At the very low conversion values (α = 0.01–0.02; Δ*T* = 76.66–133.77 °C), extremely low negative values of both *E_a_* and *logA* were identified (Figure 5a–d). This behavior is not uncommon, considering that the surface binding moisture was released very quickly and there is almost no real potential barrier, which follows from an important fact: the first endothermic event visible at both, the DTA curve (Figure 4c) and the DSC curve (Figure S2) corresponds to water desorption. Namely, this event occurs at different temperatures depending on the degree of substitution (DS) of the sample. The variance in values of the desorption peak in polymers can be explained by the different water-holding capabilities and polymer-water interactions. Results clearly show that our sample with high DS has a low value of the desorption temperature (from Figure 4c, it is 69.76 °C, while from Figure S2, it is even 27 °C). By means of acetylation, the OH groups are replaced by the acetate groups, preventing the strong interaction of the hydroxyl groups present in the CAc chain via hydrogen bonds. In this way, the residual moisture seems to be isolated and leaves the system very quickly, without the existence of any energy barrier to slow it down. In contrast, for pure cellulose, these interactions would be exceptionally intensive.
(b) With an increase in conversion and temperature (α = 0.03–0.08; Δ*T* = 147.46–185.20 °C), we enter the reaction zone, where the decomposition of the plasticizer takes place. In this conversion range, the *E_a_* value (considering the FR method (Figure 5a)—a similar situation is valid for the VY method), increases from 18.685 kJ/mol to 100.054 kJ/mol, whereas for TA (triacetin) plasticizer, the activation energy for its evaporation is found to be 65.12 kJ/mol [67]. It should be mentioned that exposure of TA in CFs to the present metals may lead to a significant change in the rate-limiting steps of gas-phase decomposition of TA, turning them into an autocatalytic scenario while lowering the nominal activation energy and changing the barrier height;
(c) By elevating the temperature and conversion values (Δ*T* = 189.31–250.66 °C, α = 0.09–0.15), it enters the reaction zone for obtaining the pyrolysis products from the thermal decomposition of TA, which takes place intensively in the gaseous phase. In the actual case, the activation energy increases from 99.311 kJ/mol up to 193.66 kJ/mol. The possible reaction pathway involving the decomposition of the TA structure is the one that represents triacetin decomposition by elimination of the acetic acid [68]. The energy required for this disruption amounts to 192.9 kJ/mol [68], which is in agreement with our results (Figure 5a). In such a reaction scheme, acetic acid can be eliminated from TA, forming prop-1-ene-1,3-diyl diacetate (note: this compound may exist in two stereoisomers (cis and trans)). However, this intermediate can further react via internal rearrangement of prop-1-ene-1,3-diyl diacetate into 2-formyl-3-oxobutyl acetate, with an *E_a_* value of 202.09 kJ/mol [68], where in our case, this value corresponds to the *E_a_* value of 202.702 kJ/mol at α = 0.16 (~254.61 °C). Also, it is possible that the intermediate decomposed into acetic anhydride and acrolein (acrolein is a notorious air pollutant) [69], with slightly higher activation energy (210.04 kJ/mol) [68]. For our data, it is the value of *E_a_* = 219.265 kJ/mol at α = 0.17 (~261.16 °C);
(d) The next reaction zone includes the thermal decomposition of the cellulose triacetate (CTA) (approx. from α 0.16 to α 0.37 for the temperature range of Δ*T* ~254.61–319.25 °C), including a raise in the activation energy (*E_a_*) from approx. 202.702/219.265 kJ/mol up to 349.922 kJ/mol (Figure 5a). Since those acetate groups can make the cellulose more stable, this requires higher energy input in order to remove them from the cellulose skeleton, so we have an increase in activation energies. Unlike cellulose, in the case of CTA, the presence of acetate substituents may influence the course of decomposition considerably by blocking the formation of 1–6 glucosan linkages. It was found that as the DS value increases, the activation energy increases progressively, requiring higher energy input to start the deacetylation process [70]. It is most probable that the first acetate group to be removed from a particular ring unit will be attached either to positions C2 or C3. If the elimination of the first acetate group was followed by the elimination of that attached to position C6, a peripheral olefinic linkage in the 5,6-position would be formed, which, being outside the ring, would not necessarily confer its instability. At this stage, we can assume that the elimination of acetate groups takes place without the disintegration of the ring unit;
(e) The next, slightly wider reaction zone of the change of *E_a_* with conversion (α) encompasses α range between α = 0.38 and α = 0.87 (Δ*T* = 320.51–363.07 °C), where there is a gradual decrease in the activation energy value, from *E_a_* = 350.603 kJ/mol to *E_a_* = 274.857 kJ/mol (Figure 5a). This phase probably includes thermally induced reactions on the skeletal chain segment via the chain-scission process. In the initial stage of this process, the tar formation may dominate, including an appearance of some kind of unsaturation arising from the loss of acetate substituents (the previous stage), which is a necessary precursor to the chain-scission process in CTA. In this case, it is most likely that the scission of β(1→4) glycosidic bond (the glycosidic scission) [71] between two rings may give 1,2-unsaturated species, which can react with phenylhydrazine molecules (formed within the tar fraction) [72]. In this case, the formation of a stable product is expected over the double bond shift and a hydro-ring closure;
(f) With further intensification of the process, in the conversion range of α = 0.88–0.93 and Δ*T* = 364.29–401.22 °C, there is a sudden drop in the activation energy value, from approx. *E_a_* = 259.279 kJ/mol up to *E_a_* = 21.664 kJ/mol (Figure 5a). In this phase, there is the formation of carbonaceous residue. Namely, in this reaction zone, the chemical fragment formed by the scission process on the glycosidic bond would probably be an oxygenated species, such as pyrylium (C_5_H_5_O), where the decarbonylation reaction dominates (releasing CO gas) [73]. These transformations probably occur above 360 °C and terminated an extensive mass loss stage in the decomposition process (Figure 4);
(g) At the very end of the decomposition process above α = 0.93 (*T* > 402 °C), there is an increase in the activation energy value (from *E_a_* = 24.245 kJ/mol up to *E_a_* = 180.950 kJ/mol (at α = 0.99)). The reason for this behavior lies in the fact that secondary decomposition of tarry material occurs, which also significantly contributes to the formation of the residue. We can assume that oxygen is present in the condensing species, so the solid residue has such features as non-graphitizing material. This can be clearly seen from the XRD analysis presented in Figure 3b for the carbonized sample, considering the appearance of planes (002), (100), and (101).

**Figure 6:**
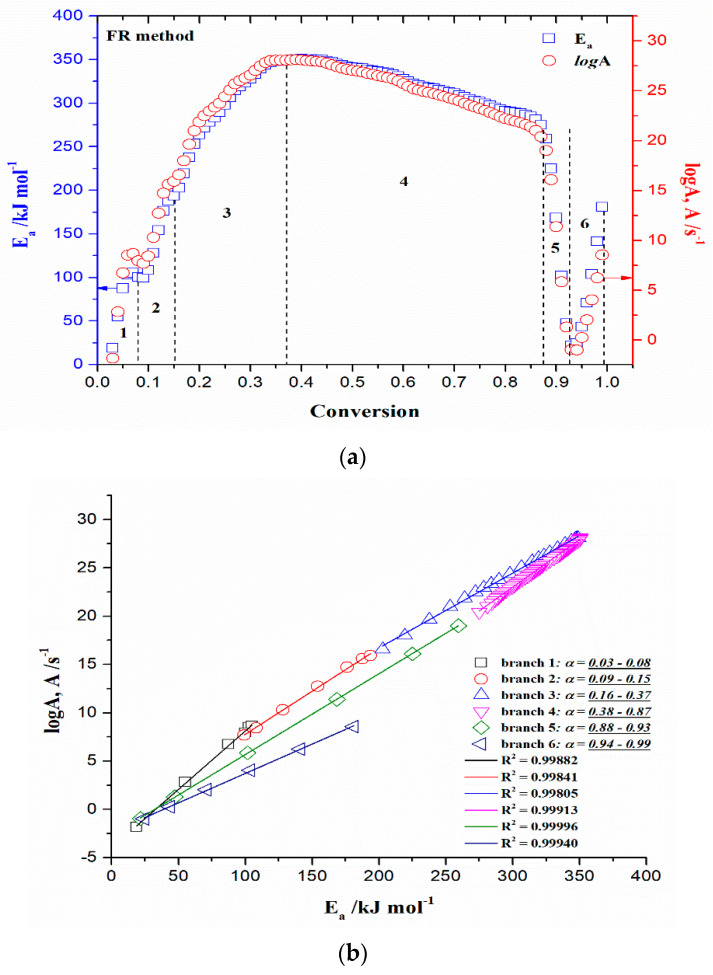
(**a**) The graphical representation of thermal decomposition stages of the r-CAcF sample, through evolutions of *E_a_*/*logA* values obtained by the FR method, under the items (see above): (b) (“1”), (c) (“2”), (d) (“3”), (e) (“4”), (f) (“5”), and (g) (“6”); (**b**) Kinetic compensation effect (KCE) “loop” formed by the variation of kinetic parameters during the thermal decomposition of the r-CAcF sample (corresponding KCE ‘branches’ were indicated, together with R^2^-values).

An overview of all the above-mentioned stages during the thermal decomposition of the r-CAcF sample is shown in Figure 6a, through *E_a_*/*logA*—conversion evolutions in a simultaneous manner, using the Friedman (FR) model-free method. In Figure 6a, the water desorption step was excluded. Figure 6b shows the relationship between the kinetic parameters (*logA* and *E_a_*), usually referred to as the kinetic compensation effect (KCE) [74]. It can be seen from Figure 6b that there is a KCE ‘loop’, which includes the decomposition process stages described above except the stage under item a) (which is neglected here).

The resulting straight-line points evidenced the presence of KCE but with a different quality of linear correlations (R^2^-value) between *logA* and *E_a_* values (Figure 6b). Namely, for branches 4, 5, and 6, there is strong compensation between kinetic parameters (R^2^ > 0.999), while for branches 1, 2, and 3, weak compensation exists (R^2^ < 0.999). The highest correlation was obtained for branch 5, related to the formation of carbonaceous residue (charring). To explain the strong compensation, especially attached to bio-char formation stages (branches 5 and 6 (Figure 6b)), one must start from the assumption of the presence of a metal catalyst in contact with the surface of the carbon material. Namely, with an increase in the temperature, sites with a high barrier to the reaction can become active. Consequently, at lower temperatures, the process rate is affected by sites with lower reaction energy barriers. Further, at higher temperatures, the process rate is affected by both types of sites, leading to a concurrent increase in the number of sites (higher *logA*) and reaction barrier as both high and low energy sites contribute to the process rate (higher *E_a_*). Probably, there are two types of sites: one that includes metal catalyst and bio-char, and another, that includes bio-char alone, without metal entity. The distribution of active sites with various energies depends on the interaction between metal(s) and bio-char. Therefore, the existence of a strong compensation arises from a specific case, viz. catalytically produced bio-char. On the other hand, the existence of weak compensation does not mean that there is no KCE; rather, it means that there is a variation in molecular ordering. This is related to a similar observation called enthalpy–entropy compensation [75], which associates the decrease in enthalpy (exothermic changes) with tauter bonding and consequently with less entropy (freedom of movement). In this context, the values of thermodynamic parameters (change of standard activation enthalpy, ΔH°, change of standard activation entropy, ΔS°, and change of standard Gibbs free energy, ΔG°) were calculated using Eyring equations [76] as follows:

(9) $$\Delta H^{o}=E_{a\;}-RT_{max},$$

(10) $$\Delta G^{o}=E_{a}+RT_{max}\cdot ln\left(\frac{k_{B}\cdot T_{max}}{h\cdot A}\right),$$

(11) $$\Delta S^{o}=\frac{\Delta H^{o}-\Delta G^{o}}{T_{max}},$$

where *T_max_* is the maximum decomposition temperature (K), *k_B_* is the Boltzmann constant (1.381 × 10^−23^ J·K^−1^), and *h* is the Planck constant (6.626 × 10^−34^ J·s^−1^). *T_max_* values are determined from the absolute conversion curves (Figure 4b) for the 1st group of peaks and the 2nd group of peaks, respectively. The activation energy (*E_a_*) and the pre-exponential factor (*A*) are calculated from Kissinger’s plots method (ASTM E 2890) [77]. The Kissinger plots constructed at various heating rates for the 1st group of peaks and the 2nd group of peaks (Figure 4b) are presented in Figures S4 and S5 (Supplementary Materials), respectively. Table 2 lists the values of *T_max_*, ΔH°, ΔG°, and ΔS° estimated for the 1st and 2nd groups of peaks situated in Figure 4b at different heating rates (*β* = 10, 20, and 30 K/min). For the calculation of thermodynamic parameters, *E_a_* and *A* values were taken from Kissinger’s method (these values are also presented in the captions of Figures S4 and S5 (Supplementary Materials)).

From the results presented in Table 2, a significant difference in the values of ΔH° between the two reaction zones can be observed. Since ΔH° represents the total amount of heat exchanged between reagents and activated complexes, the positive values in both cases indicate that external energy needs to be absorbed to lift these reagents to their transition state. However, for the 2nd group of peaks, this need is much greater. Otherwise, the ΔH° value depends on the composition of the final products obtained in the corresponding reaction zones during the pyrolysis of the r-CAcF sample. This can be related to the breaking of previous ones and creating new chemical bonds. For example, the dissociation energy needed for breaking the secondary C-C bond in the reactant amounts to 355 kJ mol^−1^ [78], so these larger amounts of energy are characterized for reactions in the second reaction zone (2nd group of peaks). Respecting their reactivity, the first zone (reactions related to the 1st group of peaks) is significantly more reactive compared to the latter. In both cases, positive and higher values of ΔG° suggest that all reactions at the transition state are non-spontaneous (Table 2). The quantity ΔS° describes the degree of disorder in the system associated with the formation of activated complexes. In the cases considered, there are negative and positive signs in the change of standard activation entropy. For the first case, the high negative entropy values suggest a more ordered system in regard to the second one (Table 2), forming an activated complex with a more organized structure compared to the initial compound (the lower the value of ΔS°, the more developed the complex, and a more ordered structure is formed) [79]. Starting from the initial compound, ΔS° values related to transformations that take place inside the 1st group of peaks are negative at all *β*’s, indicating that the structure of the activated complex from the precursor is more organized. Considering the entire pyrolysis process, there are both negative and positive ΔS° values, which indicates that the thermal decomposition of r-CAcF reflects a distinct kinetic/thermodynamic complexity. For detailed disclosure of the entire reaction mechanism of r-CAcF thermal decomposition, the use of model-based (model-fitting) kinetic analysis (this approach has none of the disadvantages that can be observed when using model-free methods due to lack of familiarity with the kinetic model function (Section SI, Supplementary Materials)) will provide us with information about the individual reaction steps that take place in a complex pyrolysis scheme (see the next subsection).

**Figure 7:**
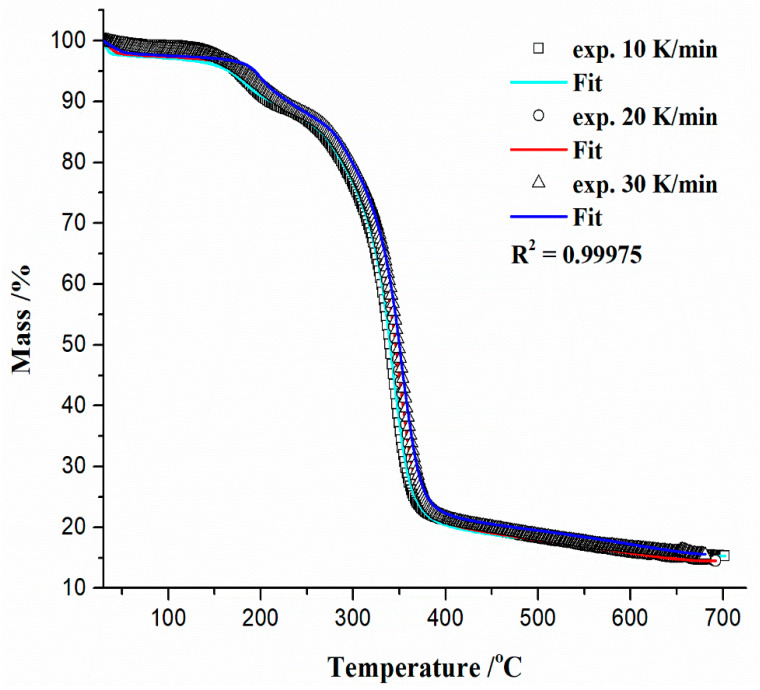
The comparison between experimental (symbols) and numerically optimized (full lines) TG-curves for the thermal decomposition of the r-CAcF sample, where numerical optimization was carried out using the kinetic parameters obtained from model-free (Friedman’s) analysis. The coefficient of fitting quality is also provided (R^2^ = 0.99975).

Finally, the numerical optimization procedure of the devolatilization process was performed based on the results of model-free (Friedman (FR)) analysis. Figure 7 shows the comparison between the experimental and numerically optimized TG-curves at different heating rates (10, 20, and 30 K/min) for the thermal decomposition process of the r-CAcF sample.

The obtained results (Figure 7) show that there is good agreement between the experimental and calculated TG-curves through the numerical optimization using model-free (Friedman (FR)) data (Figure 5a,c). These results confirm the high accuracy and reliability of the obtained kinetic parameters using isoconversional (model-free) analysis. Kinetic parameters derived from this approach can be used as the initial parameters for searching for the most optimal reaction models that realistically describe individual reaction steps in the complex pyrolytic scheme.

##### 3.3.4.2. Model-Based (Model-Fitting) Results

In order to obtain more detailed information about the mechanism of the pyrolysis process, the model-based (model-fitting) approach was used. Using the multivariate non-linear regression method (MVarNLRM) through optimization of models (Table S1, Supplementary Materials), the best mechanistic scheme for the investigated process was proposed.

Based on the searching procedure in the model-based approach, the best statistically derived mechanistic scheme was obtained, together with kinetic parameters and the contribution of individual steps, through the following scheme, designated as the q:, model, with reaction functions as follows: A*n*(*n*-dimensional nucleation (Avrami-Erofeev)) *C_nm_* (*n*-th order and *m*-power with autocatalysis) Nk (A*n* + H-L) (Nakamura crystallization) R3 (Phase boundary-controlled reaction (contracting volume, 3D)) (Table S1, Supplementary Materials). The proposed process scheme contains two individual single-step reactions and one consecutive reaction step, presented as follows:

(12) $$A\;\overset{1.1\;(An)}{\to }\;B\;$$

(13) $$C\;\overset{2.1\;(Cnm)}{\to }\;D$$

(14) $$E\;\overset{3.1\;(Nk)}{\to }\;F\;\overset{3.2\;(R3)}{\to }\;G$$

where *A*, *C*, and *E* are the reactants, *F* is the intermediate species, and *B*, *D,* and *G* represent the final products. The sequence of equations listed above does not have to strictly follow the order of changes along the entire TG-curve going from left to right (Figure 7) but performs the fitting according to appropriate reaction temperature segments. In the following sections, we will discuss each of the reaction stages in the order of their occurrence at appropriate temperature intervals during the pyrolytic process. Table 3 lists the calculated values of the kinetic parameters, kinetic exponents, reaction orders, and contribution of each reaction step (Equations (12)–(14)) to the entire pyrolysis process of the r-CAcF sample, using MVarNLRM on the obtained thermo-analytical data.

**Figure 8:**
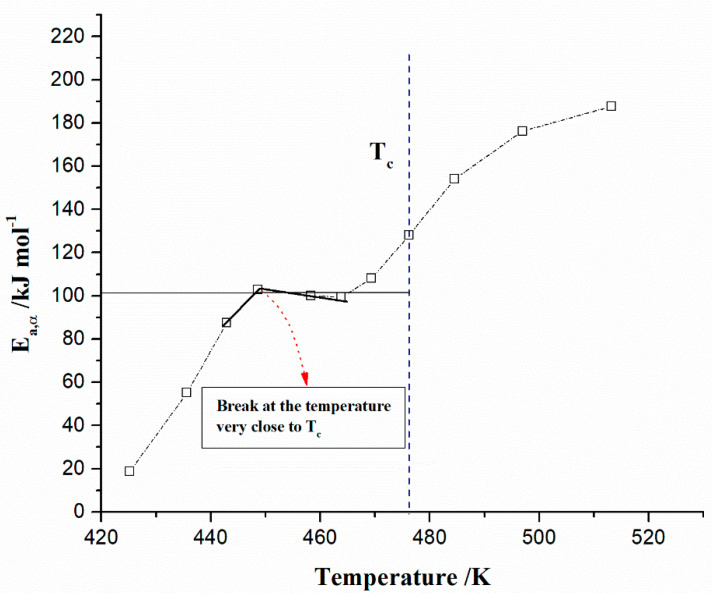
*E_a_*_,α_(*T*) dependency determined from model-free (FR) analysis extracted for a narrow temperature range around *T_c_*.

**Figure 9:**
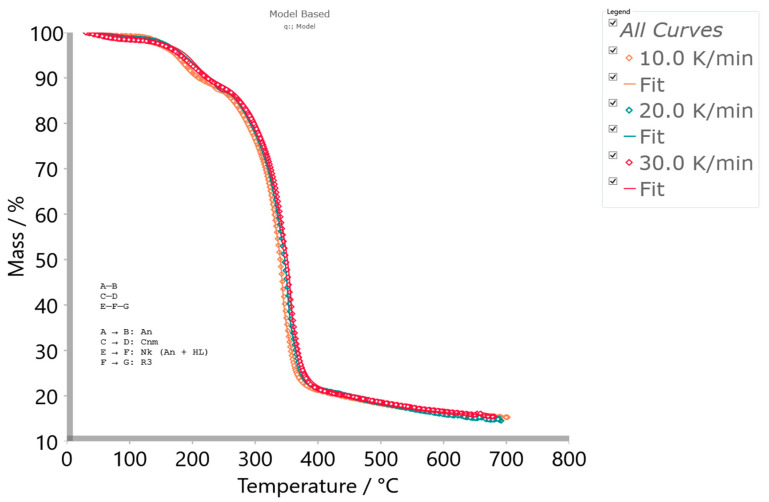
Experimental (colored symbols) and simulated (colored full lines) of the pyrolysis process of r-CAcF, considering q:, model scheme (Equations (12)–(14)). The goodness of the fit of a model R^2^ = 0.99987.

**Figure 10:**
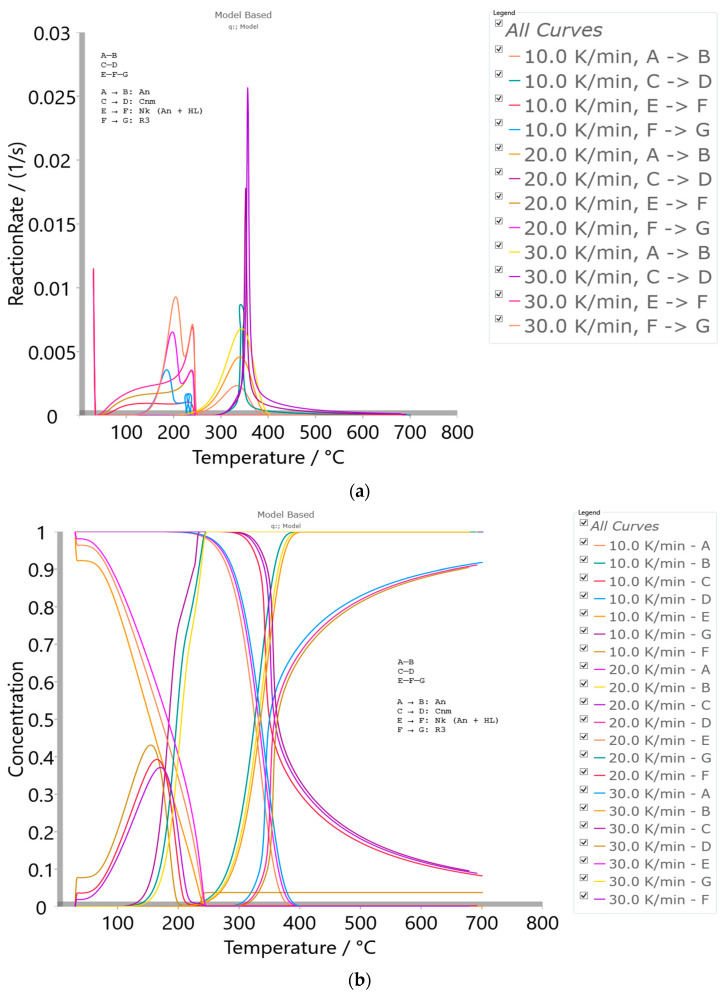
(**a**) The reaction rate (absolute) (1/s) vs. temperature (°C) for individual reaction steps within the q:, model scheme, for each of the heating rates used in the r-CAcF pyrolysis process; (**b**) Concentration (normalized) evolution of reaction species in relation to pyrolysis temperature, where the distribution of concentrations refers to each reaction step in the q:, model scheme, for each of the heating rates used.

A mechanistic interpretation of the reaction scheme proposed above is given in the following items:

(a) The consecutive reaction steps occurring via Equation (14), include the r-CAcF pyrolytic stage in the temperature range of approximately Δ*T* 30–260 °C, where the reaction step 3.1 is described by Nakamura crystallization kinetics, with a concentration equation in the form:
  (15) $$\frac{d\left(e\;\to f\right)}{dt}=A\cdot n\cdot e\left\{{\left[-ln\left(e\right)\right]}^{\frac{\left(n-1\right)}{n}}\right\}\times exp\left[-\frac{U^{*}}{R\left[T-\left(T_{g}-30\right)\right]}\right]\times exp\left[-\frac{K_{G}\left(T+T_{m}\right)}{2T^{2}\left(T_{m}-T\right)}\right],$$
  where *A* is the pre-exponential factor, *n* is the Avrami dimension parameter, U* is the activation energy of the segmental jump (the activation energy necessary for the macromolecules to diffuse to the crystal phase in the melting state) (this parameter has universal value of 6.300 kJ mol^−1^), (*T_g_* − 30) = *T*_∞_ represents the hypothetical temperature below which the molecular chain ceases motion, which is commonly defined as (*T_g_* − 30) (K) (taken 30 K below the glass transition temperature, *T_g_*), *K_G_* is the nucleation parameter (in a function of the surface free energy; represents an activation energy of the nucleation for a crystal with the critical size), which relate to the fold and lateral surface energies. In the second exponent term on the right-hand side of Equation (15), there are marking terms that must be disclosed, such as Δ*T* = *T_m_* − *T* which represents the undercooling, while *f* = 2*T*/(*T* + *T_m_*) is the correction factor [80].
  The Nk (Nakamura) reaction model contains a combination of the Avrami nucleation model inside the Arrhenius behavior, and non-Arrhenius behavior expressed through the Lauritzen–Hoffman (L-H) nucleation theory. Since Equation (15) has two exponents, the temperature dependence of the growth rate is obviously non-Arrheniusian. The proposed kinetic model can be expressed in the general rate–law equation form via conversion (α) quantity (Section SI, Supplementary Materials), such as:
  (16) $$\frac{d\alpha }{dt}=Af\left(\alpha \right)K\left(T\right)=An\left(1-\alpha \right){\left[-ln\left(1-\alpha \right)\right]}^{\frac{\left(n-1\right)}{n}}\cdot exp\left[-\frac{U^{*}}{R\left(T-T_{\infty }\right)}\right]\cdot exp\left(-\frac{K_{G}}{T\cdot \Delta T\cdot f}\right),$$
  where this model is approximately defined for the temperature range between *T*_∞_ and *T_m_*. The non-Arrhenius approach based on the Hoffman–Lauritzen theory works for the complete crystallization range, where the temperature-dependent rate constant *K*(*T*) may involve the rate constant in the form *K*(*T*) = *A*·*exp*(−*E_a_*/*RT*), which obeys the Arrhenius approach, with even negative values of the activation energy (*E_a_* < 0) (works in the small *T*-range, below a melting temperature).
  If we look at the experimental heating temperature range of the examined sample, which is approximately between 30 °C and 260 °C, and consider results from the model-free analysis, the above-established model encompasses water desorption, plasticizer initial vaporization, formation of plasticizer decomposition products, as well as parts of the deacetylation process (items under (a), (b), (c), and (d) in Section 3.3.4.1). In that case, kinetic parameters obtained from model-free analysis but related to the actual interpretation of the proposed kinetic model in the considered *T*-range should be taken as apparent, as should the parameters related to the Arrhenius approach, because it is limited in the small temperature range, just below melting temperature (as if we were to observe that part of the process under isothermal conditions). Consequently, Nk parameters listed in Table 3 for reaction step *E* → *F*, can be considered to have their own effective values independent of those from the model-free analysis.
  In the current state of the process, considering reaction step *E* → *F*, in the case of plasticized CAc, it should be assumed that there is a two-phase system, namely, a CTA-rich phase and a plasticizer-rich phase. The volume fractions of separated phases can be strongly related to the plasticizer content of the CAc system. Since CAc represents a semi-flexible polymer, it has a large persistence length. Consequently, it may be that a specific concentration fluctuation exists, which can be related to the main cellulosic chain; apropos, the plasticizer is expelled from a localized CAc-rich domain, e.g., the bundle of cellulose chains, and then dispersed in another localized domain because of the main chain behavior. The final result can be linked to a degree of plasticizer expulsion. Namely, if the plasticized CAc is a partially miscible system, then the phase domain will be either a CTA-rich phase or a plasticizer-rich phase. We previously ruled out this possibility based on the observation of results in Section 3.3.2, supposing a heterogeneous dispersion of components. Based on the higher DS value (~2.8), it can be assumed that the present amount of plasticizer improves segmental mobility substantially, making crystal perfection and the formation of new crystals easier, favoring the crystallization process. Hence, in the above-indicated temperature region, the development of crystallinity in thermally treated CTA induced by plasticizer can be expected.
  The reaction step *E* → *F* within the consecutive sequence described by Equation (14), can be attributed to the isophase transitions of cellulose occurring below thermal decomposition temperature [81]. These transitions depend on the estimated glass transition temperature values. Namely, it was also found here as the “second” *T_g_* value (*T_g_* = 10 °C (Table 3)), which differs from the experimentally confirmed value (*T_g_* = 182.68 °C (Figure S3)), and its determination was difficult because it is outside the given experimental range, so it was founded theoretically. This is to be expected because linear amorphous polymers with a simple structure typically have one glass transition temperature, but polymers with complicated structures can have multiple isophase transitions. In a concrete situation, these transitions are linked with abrupt changes in the relaxation state of non-crystalline domains. Considering these facts, we have two *T_g_*’s, one located at *T_g_* = 10 °C and the other located at *T_g_* = 182.68 °C, both below the expected temperature of the thermal decomposition. This “second” *T_g_* value is typical for a plasticized cellulose derivative sample. With an increase in plasticizer content (wt.%), the *T_g_* can be drastically lowered. The detection of isophase transition can be performed through the ratio *T_g_*/*T_m_* = 0.66 [82,83], connected to the cellulose macromolecule. In our case, the ratio of *T_g_*/*T_m_* = 0.71 was obtained (deviation for 0.05), which is the characteristic value for cellulose derivative, so *T_g_* = 182.68 °C represents the primary glass temperature (*T_g_*_1_) for the r-CAcF sample. For pure cellulose, this glass temperature lies in the range of 217–227 °C [84]. For cellulose, this refers to the primary *α*_1_ glass transition [85]. In the actual case, the “second” *T_g_* (*T_g_*_2_) occurs at a much lower temperature, indicating the strong plasticization effect of triacetin (TA), which is manifested via an important decrease of *T_g_*_2_. So, the decrement of *T_g_*’s (Δ*T_g_*) is very important in the DS series with a higher value of the degree of substitution and a high content of plasticizer.
  In the considered situation, with a significant increase in the plasticizer amount, the probability of the occurrence of *α* transition (*α*-relaxation) is extremely large, moving it towards lower temperatures [51]. Therefore, the considered step *E* → *F* can be attributed to the glass-to-rubber transition of the sample, and it is linked with motions of the CTA-rich phase in a partially miscible system (so, anyway, the partially miscible system is not excluded (Section 3.3.2), because there are two *T_g_*’s (for TA (wt.%) ≥ 40%)). The occurrence of *T_g_* at low temperatures for plasticized CAc sample (*T_g_* ~10 °C) is proof of *α* transition phenomenon, which may suppress *β*-transition [52].
  Considering parameters in Table 3 for the Nk model, there is a negative value of *K_G_* making the second exponential term in Equation (16) (the nucleation term) increase (the nucleation control during transformation), where Avrami’s dimension parameter of *n* = 0.404 suggests the one-dimensional growth of nuclei, controlled by diffusion (primary crystallization). This event may occur at temperatures up to *T* ≈ 150 °C, where the influential presence of the plasticizer can accelerate the dynamics of CTA crystallization (CTA nucleation is influenced by the presence of plasticizer acting as “diluent” for the CTA-TA blend). It should be noted that nucleation is an initial step in this process, in which crystalline structures begin to form within the polymer matrix. It is a crucial step in the crystallization process because it determines the number and size of crystalline domains that will form. Crystalline domains are regions within a material that are composed of regularly arranged and organized molecules. The number and size of these domains can dictate the properties of a given material. When nucleation is the rate-determining step, the activation energy is expected to be negative (Table 3). This is because the nucleation rate is sensitive to undercooling (the difference between the temperature of the polymer and its crystallization temperature), and the activation energy for nucleation is typically lower than that for growth.
  In addition, within the spherulites growth process, the presence of metal particles in the r-CAcF sample could act as the nucleating center of CTA and create crystal interfaces, which reduced the spherulite nucleus size and accelerated the crystallization process of CTA. The negative and large value of the nucleation parameter (*K_G_*) (Table 3) supports the presence of metal particles, reducing the energy needed to create a new crystal surface and accelerating the crystallization rate of CTA. However, if the amount of metals present in the r-CAcF sample is large, then the particles can easily produce aggregates (they aggregated together), thus reducing the number of nucleating centers and new crystal surfaces, which can have a certain inhibitory action on the crystallization process. On the other hand, *logA* has a negative value (=−1.233), giving a negative sign over the entire right-hand side of Equation (15) (and also Equation (16)), so this quantity may control the temperature dependence of the nucleation rate, related to structural dynamics. In that context, the overall crystallization rate then decreases with temperature, so as the temperature progresses, the concentration of the “crystallized” product (intermediate “*F*” (Equation (14))) can decrease. Consequently, this negative value is in agreement with the anti-Arrhenian behavior observed for melt crystallization, i.e., the crystallization rate decreases with an increase in temperature [86]. Therefore, the *E_a_*—conversion dependency obtained with the model-free isoconversional methods (Figure 5a,b) is close to the calculated data for the next reaction step (*F* → *G* (Table 3)), but some discrepancy for the *E* → *F* step is attributable to an additional mechanism, which is not taken into account in the Lauritzen-Hoffman (L-H) theory (this additional mechanism represents *n*-dimensional Avrami’s nucleation and growth model (A*n*)). Considering the reaction step *E* → *F*, the contribution of this step to the entire pyrolysis process of the r-CAcF sample is 7.3% (Table 3).
(b) The next reaction in a consecutive series represents step 3.2 (Equation (14)), described by the phase boundary-controlled reaction and presented through the concentration equation in the form:
  (17) $$\frac{d\left(f\to g\;\right)}{dt}=A\cdot 3f^{\frac{2}{3}}exp\left(-\frac{E_{a}}{RT}\right).$$
  This model represents changes in the crystallization mechanism, which is dependent on the temperature, elucidating the transition from a less ordered (usually called *α*^’^-transition) into an ordered crystalline phase (usually called *α*-transition). Namely, the first one is formed at lower temperatures (*T_c_* < 204.12 °C), and the second one is formed at higher temperatures (*T_c_* > 204.12 °C). Since the change in the crystallization rate can be generally correlated with the change in crystallization mechanism, in that case, an atypical depression of the crystal growth can appear on the occasion of this transition. Namely, the *E_a_*_,α_ – *T* dependence computed from the model-free (FR) approach (FR) (Figure 8) shows a break at the temperature near the critical temperature (*T_c_*), indicating the assumption of the change in crystallization mechanism, very close to *T_c_*. The observed *E_a_*_,α_(*T*) variation is possible only when the parameter *n* (Table 3) is replaced as the fitting parameter, which is a function of temperature (*n*(*T*)). Only in that case, the combination of A*n* + L-H models is valid for the physicochemical description of the previously considered reaction step (*E* → *F*).
  The transition that describes the previously analyzed reaction step (*E* → *F*) includes the transformation of cellulose I crystallites (described by the Nk (A*n* + L-H) model (Equation (15)) (slower crystallization (impact of L-H linear growth rate) with less ordered crystallites)) into a more stable form, cellulose II, which has a more ordered structure (formation of ordered crystallites) [87]. This is described by the reaction model expressed in Equation (17). The actual process occurs rapidly (or ‘faster’) on the surface of the solid product. The reaction is controlled by the resulting reaction interface progressing toward the center. As the reactant (in this case, the “intermediate product” *F* (Equation (14))) is transformed, the crystal volume must grow at a rate proportional to the velocity of the phase boundary, raised to the power of dimensionality of the crystal growth (*n* = 3 (Equation (17)), three-dimensional shape). In the current case, the dimensionality of cellulose crystallites probably rises into the cylindrical features, which obviously indicates a change in the reactant particle sizes, and this will alter the transformation reaction rate. With the increase in the rate of transformation of cellulose I into cellulose II, the presence of alkali metals [88] and the deacetylation process can be strongly affected. This step is characterized by an activation energy of 101.876 kJ mol^−1^ (Table 3) (this value obtained by model-based analysis was marked in Figure 8 with a horizontal solid line). Considering the reaction step *F* → *G*, the contribution of this step to the entire pyrolysis process of the r-CAcF sample is 6.8% (Table 3). Confirmation of these transformations is shown by the results in Table 2, where negative activation entropies were obtained, indicating that a much more organized structure is formed. In this context, the reaction pathway described by the Equation (14) represents the synthesis route for the production of cellulose allomorph (cellulose II), where the main intrachain hydrogen bond represents an H-bond with O6–H•••O2 position [89]. The major driving force in the actual case represents the entropy, where, if we pay attention to the results presented in Table 2, the high negative values of ΔS° (for temperatures between 447.18 K and 471.63 K) affect the decrease in the values of ΔH°, because the higher the temperature, the greater the product *T*·ΔS° will be.
(c) The single-step reaction pathway described by Equation (12) (step 1.1) is characterized by an *n*-dimensional nucleation model (Avrami-Erofeev (Table S1, Supplementary Materials)), through concentration equation in the form:
  (18) $$\frac{d\left(a\;\to b\right)}{dt}=Ana{\left[-ln\left(a\right)\right]}^{\frac{\left(n-1\right)}{n}}exp\left(-\frac{E_{a}}{RT}\right),$$
  which takes place in the temperature range of approximately Δ*T* ~260–400 °C, with kinetic parameters values of *E_a_* = 335.470 kJ mol^−1^, *logA* = 27.079 and a dimensionality parameter of *n* = 0.339 (Table 3). The observed reaction step occurs in the high-temperature region, where the start of the parallel reaction step 2.1 (Equation (13)) also takes place in this region. These steps probably manifest a partial overlap in behavior. The reaction step described by the mechanism via Equation (18) can be attributed to the main decomposition stage, where the breaking of important bonds in the CAc structure occurs. In the above-indicated temperature range, the main decomposition of cellulose appears [90], where we have the largest mass loss of the sample (Figure 4).
  The A*n* model indicates an “acceleratory” type of model, such as nucleation related to the scission mechanism. This mechanism includes the chain scission and volatilization of fragments to obtain gaseous products. The obtained activation energy (for the reaction step described by Equation (18)) of 335.470 kJ mol^−1^ was obtained (Table 3) and corresponds to levoglucosan (LG) production (~327.47 kJ mol^−1^ of absorption of the heat) according to the optimal pathway [91]. The mechanism involves the hydrogen atom of the C_6_–OH hydroxyl group transferring to the glycosidic bond in cellobiose, and the glycosidic bond cooperatively cleaves. At the same time, C6–O• connects to C1 to produce LG. It should be noted that the scission of glucosidic linkages may lead to mono- and di-functional radicals. This stadium produces H_2_O and CO_2_ gas by cracking [92], while the cellulose structure contains abundant—HCOH fragments, whose cracking may generate many radicals such as •H, •CO, or •CH and •OH, which can further react to form gaseous products. The analyzed step represents the formation of the major fraction of volatile tar (LG, 1,6-anhydroglucose). Equally, transglycosylation can yield cellobiosan and cellotriosan with considerably lower yields compared to the LG [93]. The compounds consisting of two or more pyranose rings are the components of non-volatile tar. The derived kinetic parameters for step 1.1 are in good agreement with the results of model-free analysis (Figure 5 and Figure 6a), but this reaction segment of the process is the slowest, so the high values of kinetic parameters (Table 3) can also be elucidated by the accumulation of nuclei in the cellulose. Since the rate of pyrolysis increases with increasing surface area of the interface between the phase of cellulose polymer and the phase of products, even a few nuclei of larger sizes can cause the pyrolysis rate to lag. The contribution of this step to the entire pyrolysis process of the r-CAcF sample is 54.8% (Table 3).
(d) The next single-step reaction pathway described by the Equation (13) (step 2.1) is characterized by an *n*-th order and *m*-power mechanism with autocatalysis (Table S1, Supplementary Materials), which takes place in the temperature range of approximately Δ*T* ~300–700 °C and can be presented with a concentration equation in the form:
  (19) $$\frac{d\left(c\;\to d\right)}{dt}=Ac^{n}\left[1+AutocatPreExp\cdot d^{m}\right]exp\left(-\frac{E_{a}}{RT}\right),$$
  with kinetic parameters of *E_a_* = 265.076 kJ mol^−1^, *logA* = 20.278, the reaction order of *n* = 13.969, *log*(*A*)_Autocat_. = 4.877, and an autocatalytic power exponent of *m* = 4.908 (Table 3). The current reaction step overlaps with the previously analyzed one, where an exothermic peak (exo_2_ (Figure 4c)) at 348.84 °C occurs. This can be related to the random dissociation of the bonds in the cellulosic chains along a fiber axis—1,4-β-glycosidic linkages. Further, a very important fact that should be emphasized is the following: the catalytic reaction step *C* → *D* represents the hydrolytic decomposition of cellulose by the catalytic cleavage of the glycosidic bonds because the glycosidic linkage in the cellulose molecules is very sensitive to acid-catalyzed hydrolysis [94]. Namely, the degree of this sensitivity varies according to accessibility (amorphous or crystalline region), type of acid and concentration, and also temperature. In our considered case, the process is characterized by a very high value of reaction order (~13.969, referring to the large number of reactive centers in the chains), as well as a higher value of power exponent (*m* ~4.908), which is directly related to the abundant production of products of the reaction.
  In this case, an acid trigger represents the subsistence of titanium dioxide (TiO_2_) (see results above) in the reaction system, which acts as a water-tolerant acid catalyst for biomass conversion [95]. The influence of temperature and humidity (the presence of water vapor traces) on the TiO_2_-catalyzed decomposition of cellulose derivatives can have a large impact. Namely, the higher temperatures and particularly high participation of H_2_O will enhance the decomposition [96]. However, this step was spread until the end of the entire pyrolysis process (the high-temperature region), which includes the formation of aromatic compounds (bio-char production). The aromatization of volatiles is initiated at approximately 380 °C, but an important fact that should be emphasized is that the structural reconstruction of the reacted system may occur at a temperature around 400 °C (Figure 4c), forming abundant C=O or (O)C–O chemical functionalities (Figure 2) in the bio-char [97]. With the increase in temperature above *T* ~500 °C, the stable stage of the formed carbon has been achieved. Established results are in good agreement with those obtained from model-free analysis (Figure 5 and Figure 6a), near the end of the considered process (around conversion of α ~0.90).
  Considering that melting (see *T_m_* value in Table 3) and decomposition processes are partially overlapped, the proposed model scheme coded by q:, (with reaction steps and the kinetic models presented via Equations (12)–(14)) describes very successfully the complete pyrolysis kinetics of the examined initial material (precursor’s). The validity of the proposed model can be seen in Figure 9, where a comparison between experimental and simulated (calculated) TG-curves for an established mechanistic scheme (Equations (12)–(14)) is shown.
  It can be seen from the obtained results (Figure 9) that there is a high reliability and accuracy of the r-CAcF pyrolysis optimized values extracted from model-based (model-fitting) analysis through the proposed q:, model scheme (the high value of R^2^ (quality of fit)). In addition to the current analysis, Figure 10a shows absolute reaction rates (in s^−1^) of individual steps as a function of pyrolysis temperature (*T* in °C), where the occurrence of each individual step according to the given heating rate is also indicated.

It can be seen that the distribution of reaction rates of species that evolves during the pyrolysis process of the r-CAcF sample (Figure 10a) fully corresponds to the process stages discussed above, where the two-moded peak, which spreads until the temperature of approximately 245/250 °C, corresponds to the consecutive reaction steps described by Equation (14), which would be expected. On the other hand, the two single-step reactions (Equations (12) and (13)) show strongly overlapped behavior, especially for a narrower temperature range, between 350 °C and 400 °C. Additionally, it should be noted that the reaction step *C* → *D* at all heating rates significantly contributes to the formation of solid (carbon) residue, especially at a high heating rate, e.g., 30 K/min. Thus, for pushing the process towards the formation of bio-char, higher temperatures are recommended (intensified over the high heating rate value), while for the high yield production of tar products, lower and/or medium temperatures are recommended here. Modification of the heating rates used is necessary, considering that reaction step *A* → *B* is the slowest when applying a heating rate value of 10 K/min (lower heating rate of the sample). The confirmation of correctness and the physically plausible pyrolysis mechanism of the investigated r-CAcF sample clearly show the results, which are presented in Figure 10b. The distribution of normalized concentrations of reaction species in the pyrolytic system at each heating rate as a function of temperature shows the correctness (proving the realistic approach) of the proposed mechanistic model.

### 3.4. Results of Carbonization Stage—Macro-Scale Experiments

This section covers the results obtained from carbon synthesis via a one-step carbonization process, including investigations of the morphology and chemical composition of CAc800(1h) sample using SEM-EDX analysis as well as the porosity analysis (BET) of the obtained material.

#### 3.4.1. Results of Characterization of CAc800(1h) Sample by SEM-EDX Analysis

**Figure 11:**
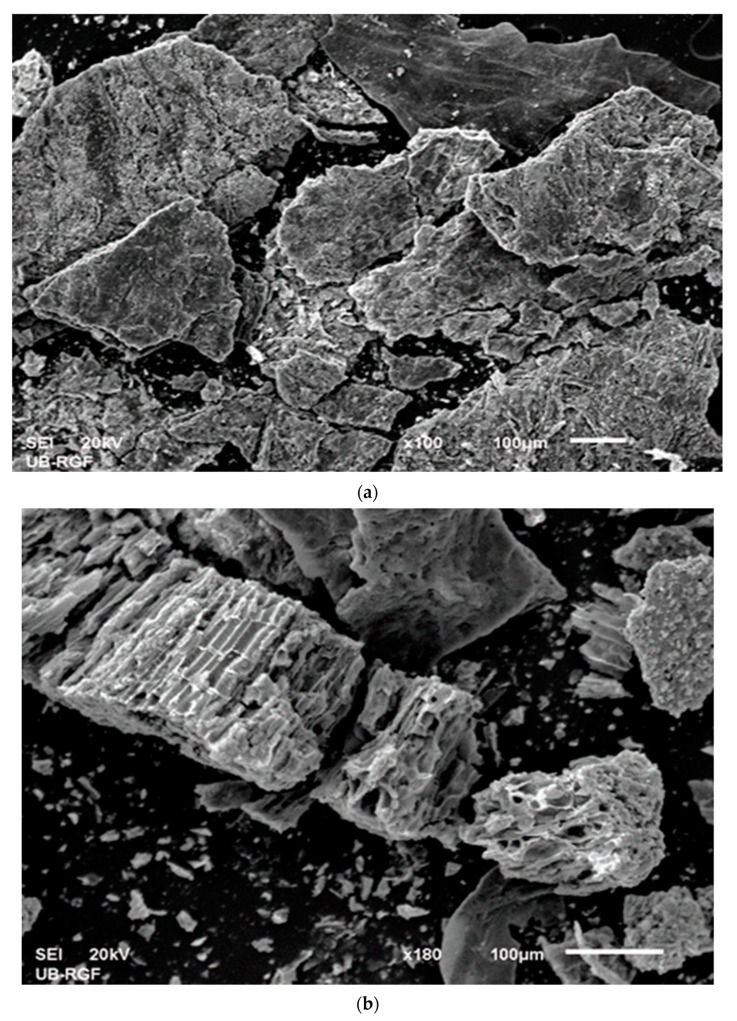

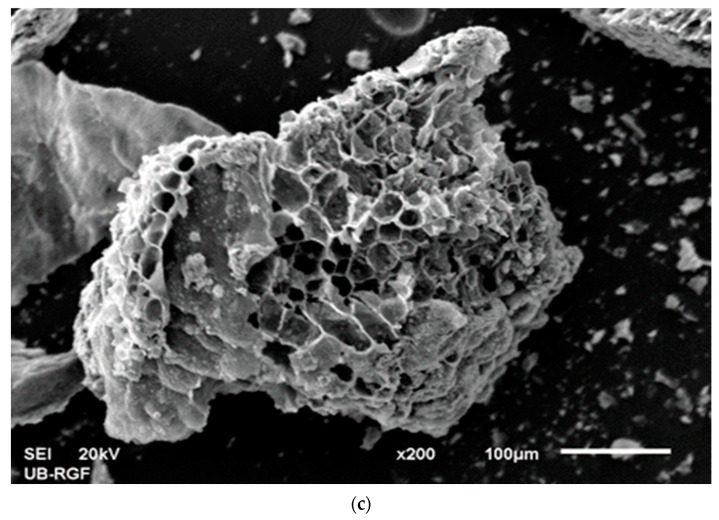
SEM images of CAc800(1h) sample at magnifications: (**a**) ×100, (**b**) ×180, and (**c**) ×200.

The morphological structure of synthesized CAc800(1h) (carbon) material was analyzed by SEM. The SEM images of the CAc800(1h) with different magnifications are shown in Figure 11a (×100), Figure 11b (×180), and Figure 11c (×200). As can be seen from the attached SEM images (Figure 11), the morphology of the obtained carbon, investigated by the SEM, is strongly dependent on the nature of the carbon’s precursor. The carbon produced from the cellulose derivative precursor shows a compact structure (Figure 11a)). There is a typical carbon monolith structure with very well-developed porosity and many large pores (Figure 11b)), confirming the successful carbonization of the precursor’s fibers. It is evident that synthesized material contains a larger number of pores with bigger dimensions, but also smaller pores too. Therefore, the obtained carbon appears to be porous, presenting many macropores, and can be considered a good sample for the BET analysis, whose results will be depicted later. On the other hand, on the CAc800(1h) sample (Figure 11), it is possible to see the existence of some white granules, which originate from calcium crystals (this can be seen better from the smoother morphological texture presented by the SEM image in Figure 11a). Namely, the calcium (Ca) melts at 842 °C, which is above the carbonization temperature that was applied. However, on the other hand, calcium (Ca) will evaporate at an appreciable rate until the temperature reaches *T* ~500 °C. At higher temperatures, there is a risk of calcium oxide (CaO) contamination, but this can be avoided as long as the temperature is controlled well below 1000 °C. Since that carbonization temperature was held at 800 °C in our case, this was prevented. In addition, potassium (K) has a low melting point (~63.5 °C), and the confirmed K chemical form in the carbonized sample represents KCl (see Figure 3b). For KCl, its melting point was 770 °C, and by involving a higher temperature, its saturated vapor pressure rises as well as its mass loss rate, causing transport resistance inside the porous medium [98]. This may form some kind of potassium coating (which can become liquid) on fibers and thus prevent fibers from reacting with each other, maintaining a fibrous structure and hindering the development of pores (upper left sequence in Figure 11c).

Furthermore, by applying high-temperature carbonization at 800 °C, the honeycomb-like porosity of the carbon material was obtained (Figure 11c). There are holes between the skeletons around each honeycomb, but bigger cylindrical bulges dominate. Consequently, the SEM image with higher magnification (Figure 11c) successfully shows the presence of macropores and porous structure on the surface of the synthesized carbon (CAc800(1h) sample). It should be emphasized that in the presence of titanium(IV) oxide (TiO_2_), the TiO_2_ can influence by the apparent decrease in particle sizes due to partial depolymerization of cellulose since, in the presence of TiO_2_, the polymer glycosidic bonds break in acidic medium and the resulting hydrolysis can be catalyzed by metal ions (see the above-presented discussion). In addition, the carbonization temperature and the heating rate play a very important role in surface morphology. It was reported [58,99] that when cigarette filters were carbonized at 800 °C for 7 h at the very slow heating rate of *β* = 1 °C min^−1^, and when they were carbonized at 800 °C for 9 h at a higher heating rate of *β* = 3 °C min^−1^, they showed a different morphology, which resulted from two different operating conditions. It seems that the higher residence time results in partial destruction of surface pores and could generate a higher specific surface area. Bearing in mind the same operating temperature and, in our case, a higher heating rate and much shorter residence time, we can expect a large porosity development at the expense of a large specific surface area.

**Figure 12:**
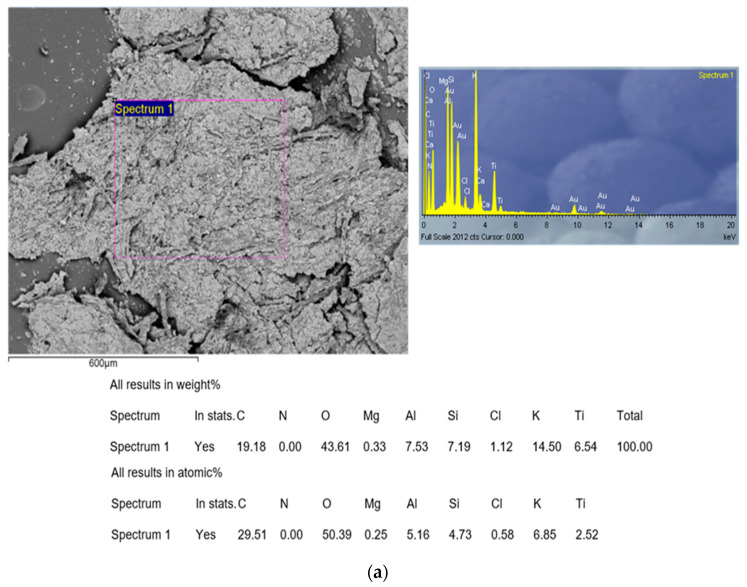

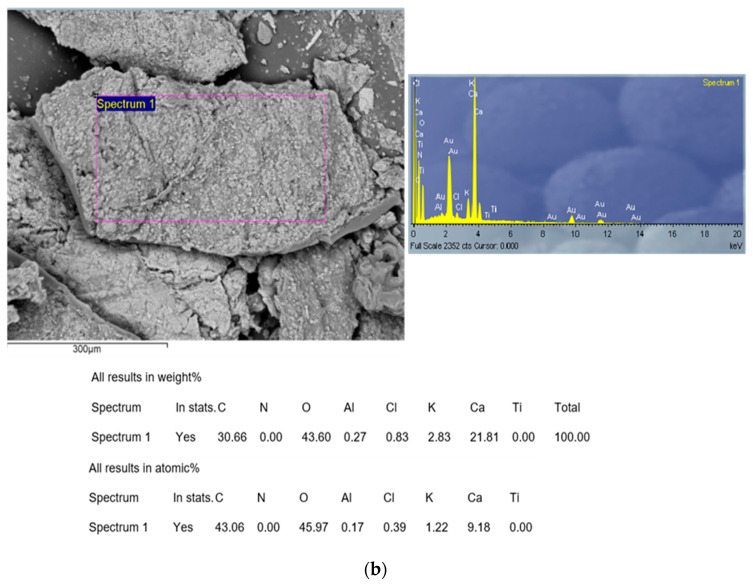
SEM micrographs and EDX analysis of (**a**) CAc800(1h) for region 1 from the SEM image, and (**b**) CAc800(1h) for region 2 from the SEM image, respectively.

To further analyze the prepared carbon material (CAc800(1h)), EDX analysis was utilized. Figure 12a,b show two EDX spectra attached to different selected regions on SEM images: region 1 (a) and region 2 (b), respectively. Both spectra give weight (%) and atomic (%) fractions of elements in the presented figure.

It can be seen from Figure 12a,b, that there is a variation in chemical composition regarding isolated regions on SEM micrographs. Considering these results, it is evident that the final porous material is composed of more than 30% (wt.%) of elemental carbon (C) and a fairly high content of oxygen, more than 40% (wt.%) (Figure 12a,b). However, it can be observed the existence of potassium and calcium particles, as well as the presence of titanium, aluminum, and silicon as heavier particle fractions from the ash. In our case, we have C/O = 0.94, i.e., C/O < 1, which indicates that synthesized carbon material (CAc800(1h)) is the oxygen-rich material. The obtained carbon still maintains a high level of potassium and chloride in the ash area (Figure 12). The potassium (K) can accelerate the decomposition through positive catalytic coupling with oxygen-bearing organic moieties during the formation of r-CAcF bio-char. On the other hand, it can improve the thermal stability of solid residue through the protection of oxygen-free organic species during the stage of r-CAcF bio-char combustion. In addition, potassium is the flame retardant element, and the higher chlorine content often causes r-CAcF to burn slowly or even be extinguished. The higher content of calcium (Ca) (Figure 12b) and the presence of magnesium (Mg) in the ash (Figure 12a) can positively contribute to the lighter complexion of their ash. Likewise, the potassium (K) may promote the cracks and elevate their rate, while the presence of nitrogen (in the synthesized sample, the N was absent (Figure 12)) may cause inhibition. So, the ash integrity of CAc800(1h) tends to be improved by a rising content of potassium and weakened by the content of chloride (Cl); however, the chloride content is less than the potassium content (Figure 12a,b). Additionally, increased content of K and Ca can have a positive catalytic effect, for example, on the Boudouard reaction (regarding the gasification), enhancing the carbon solid residue that was produced. It should be noted here that the values obtained in Figure 12 do contribute to the CB-filter-derived carbon itself but also originate from cigarette smoke constituents trapped throughout the CAc filter. Metals that were detected can originate from additives that are regularly added to tobacco, which was left behind in CBs. K is also present and is usually added in the form of potassium sorbate in the tobacco while the presence of Ti (attributed to TiO_2_) is another tobacco additive in the cigarette industry [100].

#### 3.4.2. BET Analysis and Porosity Investigation Results

**Figure 13:**
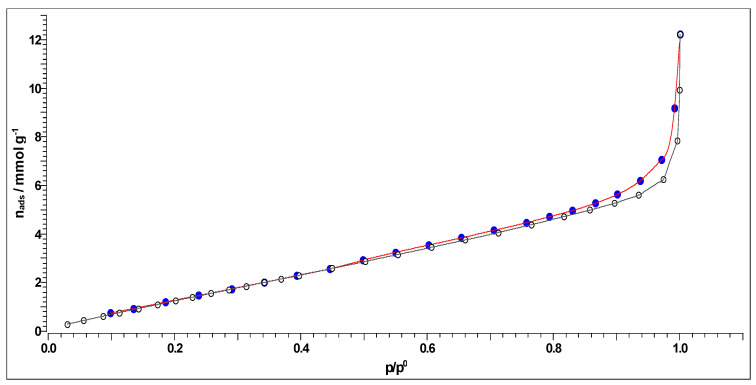
CAc800(1h) isotherm (adsorption (empty black circles))—desorption (blue solid circles) curve) at 77 K (N_2_).

BET (Brunauer–Emmett–Teller) analysis is often used to measure the specific surface area of carbon materials. The isotherm that we obtained for the sample CAc800(1h) is shown in Figure 13.

The obtained isotherm can be classified as type IV with the presence of an adsorption hysteresis loop in terms of IUPAC classification, indicating the presence of mesopores with a significant contribution from macropores [101]. This is evident from Figure 13, from the sudden increase in the adsorption quantity of nitrogen in the isotherm when *p*/*p*_o_ → 1. It seems that the surface openings have not been blocked at the carbonization temperature of 800 °C by an intermediate melt, which is often created at higher temperatures, resulting in a decrease in the BET surface area. This confirms evidence that the operating temperature of 800 °C has possibly been high enough to decompose and evaporate an intermediate melt, which can be formed by condensation of higher molecular weight (MW) molecules on the surface of the carbon material at lower temperatures. In addition, there is no deviation of adsorption and desorption branches at low (and lower) relative pressures (plots are matching each other), so any notable pore shrinkage does not exist. The hysteresis loop that appears in Figure 13 belongs to the type H1 loop (*p*/*p*_o_ > 0.8) (with larger mesopores, >4 nm) found in materials that exhibit a narrow range of uniform mesopores [102], where the narrow loop is a clear sign of delayed condensation on the adsorption branch. Besides, the hysteresis loop occurring in Figure 13 is a typical example of capillary condensation that is delayed due to the existence of metastable adsorption fluid in the adsorption branch, while evaporation occurs through equilibrium in an open pore [103]. The actual hysteresis was founded primarily in ordered mesoporous materials with uniform cylindrical pores or ordered three-dimensional pore networks [104].

**Figure 14:**
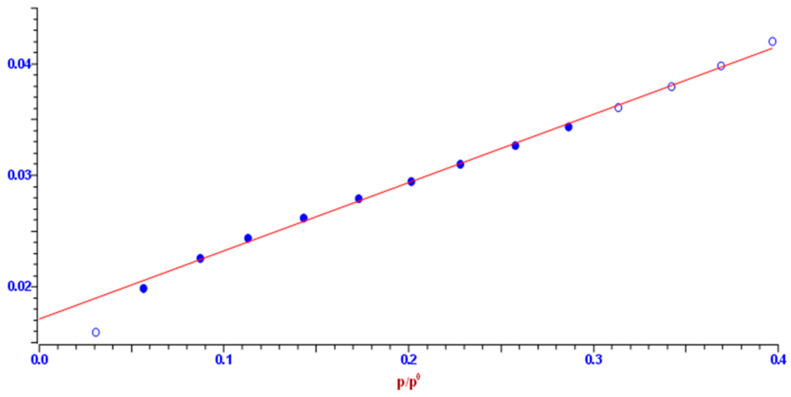
BET two-parameters line plot for CAc800(1h), where the linear regression was conducted in the *p*/*p*_o_ range from 0.05 to 0.3 (the blue fulfilled circles) (R^2^ = 0.99762).

Results obtained from surface area analysis using the BET two-parameter line method (Figure 14) for CAc800(1h) sample are summarized in Table 4. The given table includes information related to monolayer volume, monolayer amount, the *C*-(BET) constant (the heat of adsorption), and the surface area.

The positive BET (*C*) constant indicates the positive intercept in the presented plot (Figure 14), which is valid for the range used in the BET equation (the *C* value suggests a moderate interaction between solid adsorbent and adsorbate). The large monolayer volume (Table 4) is an indication of mesoporous material presence, whereas the small monolayer volume is typical for microporous materials. The acquired surface area of 55.646 m^2^·g^−1^ is produced at the expense of a marked increase in the contribution of pores with larger dimensions.

**Figure 15:**
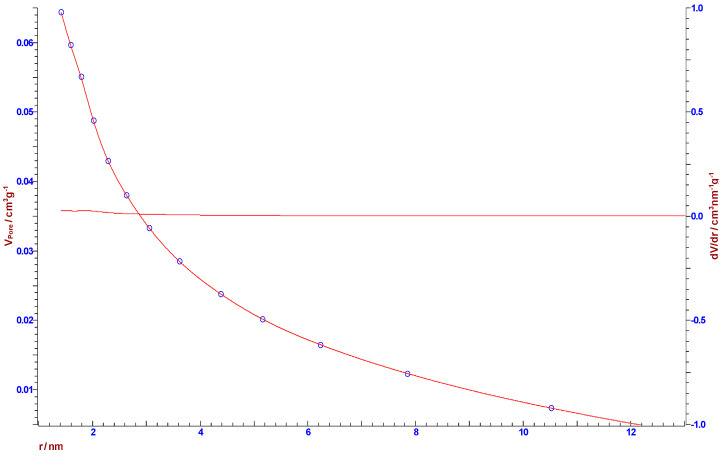
The pores distribution (d*V*/d*r*: ―○― (right *y*-axis)) and the pore volume (― (left *y*-axis)) for CAc800(1h) sample, obtained from Barrett–Joyner–Halenda (BJH) model.

Figure 15 shows the plots of pores distribution (d*V*/d*r*) and pore volume (*V*) derived by the Barrett–Joyner–Halenda (BJH) model, based on calculations of the used desorption branch from *p*/*p*_o_ = 0.3 to *p*/*p*_o_ = 0.95.

Table 5 lists the values of median pore radius, maximum pore radius, cumulative pore volume, cumulative pore area, and the pore classification with their percentage contribution, obtained from the BJH model with the de Boer standard isotherm.

It can be seen from Table 5 that the median pore radius exhibited a value of 3.1695 nm, indicating mesoporous material, where a pore radius (maximum) of 1.4191 nm may be suggested for the presence of micropores. The primarily mesoporous materials may consist of oxides, such as TiO_2_ and SiO_2_, or combinations of metal oxides, but mesoporous carbon can be synthesized [105]. Most commonly it is used to use a micellar solution and grow oxide walls around the micelles, where some inorganic salts such as metal chloride salts (e.g., KCl) can be used [105]. In the presence of rod micellar structures during the viscous crystal mechanism, in the presence of silica (or silica-like compounds) (Figure 3b), a hexagonal structural pattern can be formed, whereby any liquid form of plasticizer at high temperature is removed, so that only mesoporous structures with metal imprints remain (Figure 11 and Figure 12). The cumulative pore area is 42.6680 m^2^·g^−1^ with mesoporous pore classes ranging up to 50 nm, a pore volume of 0.0374 cm^3^·g^−1^, and the high volume contribution of mesopores of 58.1% (Table 5).

To determine the micropore volume and external surface area, the *t*-plot (Lippens and de Boer) method was used. From the constructed *t*-plot (Figure S6 (Supplementary Materials)) (for linear regression in the *t*-range from 0.3 nm to 0.6 nm), the micropore volume can be found from the intercept of the extrapolated first linear region of the *t*-plot and *y*-axis, and the external surface area (the surface area from meso- and macropores and the true external surface) can be determined from the slope of the second linear region.

The determined micropore volume has a negative value and amounts to −0.023 cm^3^·g^−1^, which means a regression in microporosity, while the mesopore’s surface area was 82.244 m^2^·g^−1^. Considering the obtained values, the *t*-plot method is very sensitive to the free space/void volume and saturation pressure errors, much more so than in the case of the BET equation (Table 4). Otherwise, a very small negative intercept (micropore volume; see above lines) can be interpreted as zero, as can the absence of micropores. Produced CAc800(1h) can be characterized as the Ti-enriched highly mesoporous carbon derived from CAc cigarette butt (CB) filters via one-step carbonization in the absence of chemical and/or physical activation. The slow pyrolysis regime of r-CAcF, guided by a complex kinetic (mechanistic) scheme, allows obtaining the main tar fraction compound (levoglucosan—LG) through the nucleation model, liberating further gaseous products such as CO and CO_2_ (H_2_O). As a consequence of these actions, fragmentations may generate furans and a series of oxygenates through dehydration (see previous discussions). By involving an acid catalyst, the autocatalysis step that takes place in the r-CAcF decomposition through acid-catalyzed hydrolysis may lead to sugars and other valuable bioproducts. On the other hand, the carbonization stage has led to the production of Ti-oxygen-rich, highly mesoporous carbons (with an acidic character).

Our results reported in this work are similar to those obtained for the pyrolysis process of used cigarette filters via microwave-assisted heating with respect to reaction pathways (but they are not identical) [106]. For the sake of comparison, Table 6 shows the BET surface area (*S*_BET_) and median pore radius *P* (nm) by the BJH model (*P*_BJH_) for the produced bio-char by microwave-assisted pyrolysis of unused cigarette filters (CF-1), unused cigarette filters treated with methanol (CF-2), used cigarette filters (UCF-1) and used cigarette filters treated with methanol (UCF-2), and the indicated porosity characteristics for our synthesized material (CAc800(1h)).

The difference in the results shown in Table 6 indicates that the microwave-assisted pyrolysis produced a much higher BET surface area of bio-char than in the case when the fixed-bed reactor was used. However, in the actual case, the pore radius developing has a more positive trend compared to CF-1 and CF-2, and it is even comparable with the values for UCF-1 and UCF-2 (Table 6). Differences can occurre in the pre-treatment of raw materials and operating parameters applied during the thermo-chemical conversion. This strongly affects the morphological appearance of the produced bio-char itself, from a much coarser and smaller irregular aggregated structure [106] to a more compacted structure of the bio-char with well-developed porosity and many large pores (pore coalescence due to the release of volatile organic compounds (VOCs) and salt melting provokes hole enlargement and reduction of bio-char surface area, ~55.65 m^2^·g^−1^) (Table 6). Based on the physicochemical characteristics of the synthesized material, it can be pointed out that CAc800(1h) could have multiple applicable purposes, such as a catalyst for gasification reactions and for wastewater treatments (however, the presence of heavy metals such as Al and Ti highlighted the fact that synthesized carbon should be used with care, and with caution in water treatment systems) [107]. For future perspective works our obtained carbon material may be doped with N (nitrogen-doped carbon) through some nitrogen sources, which would serve as the effective adsorption system for carbon dioxide (CO_2_) capture (CO_2_ capturing would be achieved through hydrogen-bonding interactions between hydrogen atoms (from CH (this chemical functionality already exists in the material synthesized here) and NH, for instance) on the carbon surface and CO_2_ molecules), considering a lower Si/Al ratio for our received carbon material (below the unity (Si/Al ~0.91)).

## 4. Conclusions

Used cigarette butt (CB) filters consisting primarily of cellulose acetate (CAc) proved to be a potentially suitable precursor for the production of valuable bio-products and an especially highly porous charred carbon through the pyrolysis process. In this paper, the proposed procedure for pyrolysis conditions enables a sustainable path for cigarette filter (CFs) recycling. The presented study was divided into two phases that examined in detail the pre-carbonization stage (devolatilization stage) by the micro-scale thermal decomposition experiments of the precursor (r-CAcF), using diverse thermal analysis techniques (simultaneous TG-DTG-DTA and DSC techniques) at different heating rates, and the carbonization stage (charring) conducted in a fixed bed reactor (as the macro-scale experiment), for producing the porous carbon. The last one was carried out via the one-step carbonization process at an operating temperature of 800 °C with a residence time of 1 h and a heating rate of *β* = 4 °C min^−1^ (CAc800(1h)). The physico-chemical characterization of the raw material as well as the carbonized sample was performed using various instrumental experimental analysis techniques, while the porosity of manufactured carbon was determined by the BET method.

The results of the kinetic analysis showed very interesting phenomena that occurred during the thermo-chemical conversion of r-CAcF, and they are listed in the following important items:

- It was assumed that acetyl groups of plasticizer (triacetin (TA)) interact with CAc through dipolar interactions and hydrogen bondings. The influence of these polar interactions may affect the position of the glass transition temperature (*T_g_*) of r-CAcF. However, weak H-bonded OH groups appear in the concert mainly due to structural changes in intrachain hydrogen bonds. It was established that sufficient thermal motion of cellulose chains may cause certain concurrent alignment of the small segment of cellulose at the primary nucleation site, which was formed by hydrogen bonding at the initial stage of the process;
- Based on the estimated value of *T_g_* from the DSC analysis, it was found that CAc present in r-CAcF has a degree of substitution (DS) equal to 2.8 (DS = 2.8). The increase in DS leads to a decline in crystallinity. A decline in crystallinity was observed, which indicates a reduction of hydroxyl groups leading to less organized chains and a decrease in intermolecular interactions via hydrogen bonding;
- The mechanistic scheme of the pre-carbonization stage of r-CAcF was kinetically complex, consisting of two reactions in the consecutive step (described by a combination of *n*-dimensional (Avrami–Erofeev) nucleation (A*n*) and Lauritzen–Hoffman (L-H) nucleation models (Nakamura (Nk) crystallization), with a three-dimensional phase-boundary model (R3), respectively), and two single-step reactions (described by *n*-dimensional nucleation (Avrami–Erofeev) (A*n*) and the autocatalytic C*nm* models, respectively);
- It was established that the first reaction within a consecutive stage can be attributed to the isophase transition of cellulose which occurs below thermal decomposition temperature. The detection of isophase transition was strongly influenced by estimated glass transition temperature (*T_g_*) values, whereby two *T_g_*’s are found: *T_g_*_1_ = 182.68 °C and *T_g_*_2_ = 10 °C. These results are related to motions of the CTA-rich phase in a partially miscible system with a high content of plasticizer. The occurrence of *T_g_* at low temperatures (*T_g_* ~10 °C) was proof of the *α* transition phenomenon. The transformation from nucleation to phase-boundary mechanism identified a change in crystallization mechanism, depending on the temperature, pointing to a transition from a less ordered (*α*’-transition) into an ordered crystalline phase (*α*-transition). Kinetic analysis of this process stage revealed a transformation of cellulose I into cellulose II (polymorphic transformation) in the presence of alkali metals in r-CAcF (which affect the increase in the rate of transformation);
- A single-step reaction that occurs in the temperature range of Δ*T* ~260–400 °C was attributed to the mechanism, including chain scission and volatilization of fragments, to obtain gaseous products. This reaction step was characterized by the formation of levoglucosan (LG) (the reaction step attached to the formation of the major fraction of volatile tar), with the release of a large number of radical species;
- Another single-step reaction (occurring in the *T*-range of Δ*T* ~300–700 °C) was attributed to the hydrolytic decomposition of cellulose by the catalytic cleavage of glycosidic bonds in the presence of an acidic catalyst (TiO_2_-catalyzed decomposition). It was found that at higher temperatures in the presence of the catalyst and with high participation of H_2_O, the decomposition can be significantly enhanced (producing value-added chemicals). This stage also permits the aromatization of volatiles through reinforcement of the previous stage because it was established that both single-step reactions show significant overlapping behavior. It was found that, through the identified kinetic scheme, abundant C=O and/or (O)C–O chemical functionalities are to be expected in the bio-char.

The thermal production of solid carbon residue was successfully carried out in the carbonization stage, and the main characteristics of the obtained product are as follows:

▪ The synthesized carbon (CAc800(1h)) showed characteristics of a macroporous material with developed mesoporosity. EDX analysis showed a certain amount of the ash, e.g., Ti, Al, and K, which were present in the final carbon product. Additionally, CAc800(1h) is highly enriched by oxygen, making it an oxygen-rich material. It was concluded that the ash integrity of CAc800(1h) may be improved by a rising content of K, and it can be weakened by chloride content;
▪ It was established that CAc800(1h) can be used for wastewater treatment. However, this should be taken with an extremely cautions approach, given the presence of heavy metals such as Al and Ti. Therefore, appropriate washing measures would then have to be taken, with the aim of removing heavy metal traces, for its sensitive applications;
▪ The production costs of CAc800(1h) are low, considering that the costs do not include chemicals because no kind of chemical or physical activation is used. Therefore, it is a fact that the production of CAc800(1h) requires little processing and consequently represents a low-cost carbon sorbent. For further applications, the increase in specific surface area through various activation techniques could be included.

## Figures and Tables

**Table 1:** Devolatilization indexes (*D_i_*) and the heat-resistance indexes (*T*_HRI_) at various heating rates (*β* = 10, 20, and 30 K/min) related to the main thermal decomposition zone of the r-CAcF sample; Values of *R_max_*, *T_in_*, Δ*T*_1/2_, *T*_5_, and *T*_30_ were also included.

*β* (K/min)	*R_max_* (%/min) ^a^	*T_in_* (°C) ^a^	Δ*T*_1/2_ (°C) ^a^	*D_i_* (%·min^−1^·°C^−3^)	*T*_5_ (°C)	*T*_30_ (°C)	*T*_HRI_ (°C)
10	15.5	226.52	37.58	5.324 × 10^−6^	172.62	315.77	126.67
20	29.6	245.62	39.70	8.673 × 10^−6^	179.99	322.17	130.00
30	43.0	249.46	43.00	1.136 × 10^−5^	181.94	325.14	131.25

^a^ Determined from the absolute conversion rate curves (Figure 4b).

**Table 2:** *T_max_*, ΔH°, ΔG°, and ΔS° values related for the first and second groups of peaks at various heating rates (Figure 4b).

1st Group of Peaks ^b^				
*β* (K/min)	*T_max_* (°C)	ΔH° (kJ mol^−1^)	ΔG° (kJ mol^−1^)	ΔS° (J mol^−1^ K^−1^)
10	174.03(447.18) ^a^	66.97	129.46	−139.74
20	189.87(463.02) ^a^	66.84	131.67	−140.03
30	198.48(471.63) ^a^	66.77	132.88	−140.18
Average	187.46(460.61) ^a^	66.86	131.34	−139.98
**2nd group of peaks ^c^**				
***β* (K/min)**	***T_max_* (°C)**	**ΔH° (kJ mol^−1^)**	**ΔG° (kJ mol^−1^)**	**ΔS° (J mol^−1^ K^−1^)**
10	342.00(615.15) ^a^	295.23	175.63	+194.41
20	350.00(623.15) ^a^	295.16	174.08	+194.30
30	353.00(626.15) ^a^	295.13	173.50	+194.26
Average	348.33(621.48) ^a^	295.17	174.40	+194.32

^a^ Values in parentheses (…), in Kelvins. ^b^ 1st group of peaks: *E_a_* = 70.69 ± 0.30 kJ mol^−1^; *A* = 1.27 × 10^6^ s^−1^. ^c^ 2nd group of peaks: *E_a_* = 300.34 ± 4.14 kJ mol^−1^; *A* = 4.98 × 10^23^ s^−1^.

**Table 3:** Model-based (model-fitting) kinetic results, for the pyrolysis process of the r-CAcF sample.

Model Scheme (q: model code):	
A—B	
C—D	
E—F—G	
**Model Reaction Steps:**	
A → B	
C → D	
E → F	
F → G	
**Step: A → B**	
**Reaction Type: An**	
Activation Energy (kJ mol^−1^)	335.470
Log(PreExp) (s^−1^)	27.079
Dimension, *n*	0.339
Contribution	0.548
**Step: C → D**	
**Reaction Type: Cnm**	
Activation Energy (kJ mol^−1^)	265.076
Log(PreExp), *A* (s^−1^)	20.278
Reaction order, *n*	13.969
Log(AutocatPreExp)	4.877
Autocatalytic power, *m*	4.908
Contribution	0.311
**Step: E → F**	
**Reaction Type: Nakamura**	
*K_G_* (×1000) (K^2^)	−58.613
Log(PreExp), *A* (s^−1^)	−1.233
Dimension, *n*	0.404
Melting temperature, *T_m_* (°C)	258.500
Temperature of Glass, *T_g_* (°C) ^a^	10.000
U* (kJ mol^−1^)	6.300
Contribution	0.073
**Step: F → G**	
**Reaction Type: R3**	
Activation Energy (kJ mol^−1^)	101.876
Log(PreExp), *A* (s^−1^)	9.214
Contribution	0.068

^a^ *T_g_* determined as the “second” glass transition temperature in undercooling conditions. U* represents the activation energy of the segmental jump (kJ mol^−1^).

**Table 4:** Physicochemical characteristics of the synthesized carbon material (CAc800(1h)) obtained from BET analysis.

Physicochemical Characteristics of CAc800(1h) Received by the BET Analysis	
Monolayer volume (cm^3^ g^−1^)	12.784 ± 0.266
Monolayer amount (mmol g^−1^)	0.570 ± 0.012
*C*	4.563
Surface area (m^2^ g^−1^)	55.646 ± 1.156

**Table 5:** The median pore radius (nm), maximum pore radius (nm), cumulative pore volume (cm^3^·g^−1^), cumulative pore area (m^2^·g^−1^), and the pore classification with their percentage contribution, for CAc800(1h) sample, using the BJH model.

CAc800(1h) Pore Characteristics from BJH Model		
Median pore radius (nm)	3.1695	
Maximum pore radius (nm)	1.4191	
Cumulative pore volume (cm^3^ g^−1^)	0.0644	
Cumulative pore area (m^2^ g^−1^)	42.6680	
Pore class	*V_pore_* (cm^3^ g^−1^) (Figure 15)	*V_pore_* (%)
From 0 [nm] to 50 [nm] classified as mesoporous material by pore classes	0.0374	58.1

**Table 6:** Surface areas and median pore radius of bio-char, produced by pyrolysis through microwave-assisted heating, and bio-char manufactured in this work by the proposed procedure.

Sample	*S*_BET_ (m^2^ g^−1^)	*P*_BJH_ (nm)	Reference
UCF-1	379.89	3.57	[106]
UCF-2	322.40	3.96	
CF-1	376.29	2.45	
CF-2	376.84	2.67	
CAc800(1h)	55.65	3.17	This work

## Data Availability

The data presented in this study are available on request from the corresponding author.

## Supplementary Materials

The following supporting information can be downloaded at: https://www.mdpi.com/article/10.3390/polym15143054/s1, Theoretical background of Model-based (model-fitting) kinetic analysis, Table S1. Kinetic model functions (in differential form of the analytical kinetic functions, *f*(α)) used in the current work, for computational procedure in the model-based (model-fitting) analysis, Figure S1. TG-curves at the various heating rates (*β* = 10, 20 and 30 K/min) in an argon (Ar) atmosphere, for devolatilization process of r-CAcF sample (designation of the main process stages (“I–IV”) and corresponding residual mass values (Δ*m_res_*) are also presented), Figure S2. DSC curve of the r-CAcF sample at the heating rate of *β* = 10 K/min, measured in the temperature range of Δ*T* = +21 °C − +377 °C. The position of melting temperature (*T_m_*) is indicated (*T_m_* = 258.50 °C), Figure S3. Determination procedure of the glass transition temperature (*T_g_*) for r-CAcF sample, Figure S4. Kissinger plot for the thermal decomposition process of the r-CAcF sample, at different heating rates (*β* = 10, 20 and 30 K/min), including the 1st group of peaks in the Figure 4b) (Kinetic parameters values: *E_a_* = 70.69 ± 0.30 kJ mol^−1^; *A* = 1.27 × 10^6^ s^−1^), Figure S5. Kissinger plot for the thermal decomposition process of the r-CAcF sample, at different heating rates (*β* = 10, 20 and 30 K/min), including the 2nd group of peaks in the Figure 4b) (Kinetic parameters values: *E_a_* = 300.34 ± 4.14 kJ mol^−1^; *A* = 4.98 × 10^23^ s^−1^), Figure S6. *t*-plot (Lippens and de Boer) method for determination of the micropore volume and external surface area, for CAc800(1h) sample (R^2^ = 0.99868). References [108,109,110,111,112,113] are cited in the Supplementary Materials.

## Nomenclature

**Abbreviations**	
CB	cigarette butt
CAc	cellulose acetate
CTA	cellulose triacetate
CF	cigarette filter
CFs	cigarette filters
ACs	activated carbons
ATR	Attenuated total reflectance
BDEs	bond dissociation energies
CBFs	cigarette butt filters
HPC	highly porous carbon
TG	thermogravimetry
DTG	derivative thermogravimetry
DTA	differential thermal analysis
DSC	differential scanning calorimetry
XRD	X-ray diffraction
XRPD	X-ray powder diffraction
FTIR	Fourier-transform infrared
SEM	scanning electron microscopy
SEM	EDX-scanning electron microscopy–energy dispersive X-ray
BET	Brunauer–Emmett–Teller
TA	triacetin
FR	Friedman method
VY	Vyazovkin method
KCE	kinetic compensation effect
ICTAC	International Confederation for Thermal Analysis and Calorimetry
r-CAcF	raw-cellulose acetate filter
*S*-BET	specific surface area determined by BET
BJH	Barrett–Joyner–Halenda
ICSD	International Crystal Structure Database
ICDD	International Centre for Diffraction Data
DS	degree of substitution
ASTM	American Society for Testing and Materials
MW	molecular weight
DMA	dynamic mechanical analysis
AGU	anhydroglucose units
CTA	cellulose triacetate
A*n*	*n*-dimensional nucleation, Avrami–Erofeev
C*_nm_*	*n*-th order and *m*-power with autocatalysis
R3	phase-boundary controlled reaction
Nk	Nakamura
MVarNLRM	multivariate non-linear regression method
L–H	Lauritzen–Hoffman
LG	levoglucosan (organic compound)
VOCs	volatile organic components
*C*	the BET constant
*D_i_*	devolatilization index (% min^−1^ °C^−3^)
*R_max_*	the maximum decomposition rate (% min^−1^)
*T_in_*	initial devolatilization temperature (°C)
*T_max_*	maximum mass loss temperature (°C)
Δ*T*_1/2_	temperature interval when the instantaneous mass loss rate equals half of the *R_max_* (°C)
*T*_HRI_	the heat-resistance index (°C)
*T*_5_	temperature at 5% of mass loss (from TG) (°C)
*T*_30_	temperature at 30% of mass loss (from TG) (°C)
*k*(*T*)	decomposition rate constant (min^−1^)
*A*	pre-exponential (frequency) factor (s^−1^)
*E_a_*	activation energy (kJ mol^−1^)
*E_a_*_,α_	activation energy at a conversion α (kJ mol^−1^)
*A*_α_	pre-exponential (frequency) factor at a conversion α (s^−1^)
*T*_α_	the temperature at a conversion α (K)
*T*_α,*i*_	the temperature at a conversion α and *i*-th heating rate (K)
*R*	the universal gas constant (8.314 J K^−1^ mol^−1^)
*T*	the absolute temperature (K)
*t*	the time (min/s)
d*T*/d*t*	heating rate (°C min^−1^)
dα/d*t*	non-isothermal (dynamic) decomposition rate (min^−1^/s^−1^)
Δ*m_res_*	the residual mass (%)
*T_g_*	glass transition temperature (°C)
*T_m_*	melting temperature (°C)
*T_c_*	crystallization temperature (°C)
ΔH°	change of standard activation enthalpy (kJ mol^−1^)
ΔS°	change of standard activation entropy (J mol^−1^ K^−1^)
ΔG°	change of standard Gibbs free energy (kJ mol^−1^)
*k_B_*	Boltzmann constant (1.381 × 10^−23^ J K^−1^)
*h*	Planck constant (6.626 × 10^−34^ J s^−1^)
*n*	Avrami’s dimension parameter (//or referred to as reaction orders)
*m*	autocatalytic parameter
(*A*)_Autocat_.	the autocatalytic pre-exponential factor (s^−1^)
*K*(*T*)	temperature-dependent rate constant (Nakamura’s rate constant) (s^−1^)
*U^*^*	activation energy of the segmental jump (kJ mol^−1^)
*T_∞_*	the hypothetical temperature below which the molecular chain ceases motion (K)
*K_G_*	the nucleation parameter (K^2^)
*f*	the correction factor in Equation (16)
**Greek letters**	
α	conversion (-)
*f*(α)	differential form of the kinetic (reaction) model (-)
*β*	heating rate (experimental) (K min^−1^)
*φ*	gas flowing rate (mL min^−1^)
**Subscripts**	
*i*, *j*	refer to independent experimental runs, performed at different heating rates
