# Supplementary material for: Upcycling of the Used Cigarette Butt Filters through Pyrolysis Process: Detailed Kinetic Mechanism with Bio-Char Characterization
Source: Polymers (Basel). 2023 Jul 15;15(14):3054. doi: 10.3390/polym15143054 (PMC10383087; doi:10.3390/polym15143054)
Supplement: Supplementary file 1 [file polymers-15-03054-s001.zip › polymers-2440319-supplementary.pdf]

Supplementary Material

Polymers

Title: "Upcycling of the used cigarette butt filters through pyrolysis process: Detailed kinetic mechanism with bio-char characterization"

Authors: Bojan Janković, Marija Kojić, Milena Milošević, Milena Rosić, Hadi Waisi, Bojana Božilović, Nebojša Manić, Vladimir Dodevski* (*Corresponding author)

Corresponding author affiliation: University of Belgrade, Department of Material Science, "Vinča" Institute of Nuclear Sciences – National Institute of the Republic of Serbia, Mike Petrovića Alasa 12-14, P.O. Box 522, 11001 Belgrade, Serbia

■ *Supplementary material content:*

<u>*Theoretical background*</u>

I. Model-based (model-fitting) kinetic analysis……………………………………………………..S-2 – S-5

<u>*Results section*</u>

Figure S1. TG-curves at the various heating rates (β = 10, 20 and 30 K/min) in an argon (Ar) atmosphere, for devolatilization process of r-CAcF sample (designation of the main process stages ("I - IV") and corresponding residual mass values (Δm_{res}) are also presented)………………………………………………………..S-6

Figure S2. DSC curve of the r-CAcF sample at the heating rate of β = 10 K/min, measured in the temperature range of ΔT = +21 °C - +377 °C. The position of melting temperature (T_m) is indicated (T_m = 258.50 °C)…….S-7

Figure S3. Determination procedure of the glass transition temperature (T_g) for r-CAcF sample………………...S-8

Figure S4. Kissinger plot for the thermal decomposition process of the r-CAcF sample, at different heating rates (β = 10, 20 and 30 K/min), including the 1st group of peaks in the Fig. 4 b) (Kinetic parameters values: E_a = 70.69 ± 0.30 kJ mol^{-1}; A = 1.27 × 10^{6} s^{-1})…………………………………………………………………...S-9

Figure S5. Kissinger plot for the thermal decomposition process of the r-CAcF sample, at different heating rates (β = 10, 20 and 30 K/min), including the 2nd group of peaks in the Fig. 4 b) (Kinetic parameters values: E_a = 300.34 ± 4.14 kJ mol^{-1}; A = 4.98 × 10^{23} s^{-1})...S-10

Figure S6. *t*-plot (Lippens and de Boer) method for determination of the micropore volume and external surface area, for CAc800(1h) sample (R^2 = 0.99868)..S-11

I. Model-based (model-fitting) kinetic analysis

Kinetic analysis and appropriate modeling of the decomposition process of the sample were made using a Kinetics Neo software package developed by NETZSCH Co. The basic concept of model-based kinetic analysis was established by Opfermann [1]. It is assumed that the material decomposes according to Eq. (S1):

$$A_{solid} \rightarrow B_{solid\ or\ liquid} + C_{gas} \tag{S1}$$

The expression of the rate d*e*/d*t*, where *e* is the concentration of reactant, is assumed to be defined through the following equation:

$$\frac{de}{dt} = k(T) \cdot f(e,p), \tag{S2}$$

where $k(T)$ is the kinetic constant, *p* is the concentration of the product, while the temperature-dependent rate constant is expressed through the Arrhenius law as: $k(T) = A \cdot e^{-E/RT}$, where *A* is the pre-exponential factor, *E* is the activation energy, while $f(e,p)$ represents the reaction (kinetic) model, usually involved in the case of the TG measurements. All reactions are assumed to be irreversible. In the case of degradation and since the evolved gases are continuously removed by the fluid flow in the TGA chamber, this is a reasonable assumption. It is also assumed that the overall reaction (Eq. (S1)) is the sum of individual reaction steps (formal or true/real steps) with constant activation energy, as generally accepted in chemistry theory. The kinetic model can then include competitive, independent and successive reactions. In the case of a multi-stage process, equations are solved using the multivariate non-linear regression method (MVarNLRM). By optimizing the models used for kinetic analysis, the kinetic parameters of each step in the decomposition model of the material can be extracted, allowing a better understanding of the thermal decomposition behavior. The model-based approach uses model-free (isoconversional) analysis results, in order to determine initial kinetic decomposition parameters, and gain insight regarding the number of steps involved, and the types of kinetic models that govern the process [2].

Kinetics Neo software includes a set of basic equations describing the macro-kinetics of the process t which is analyzed. Each stage of a process can correspond to one (or several) of the equations listed in Table SI.1. The type of $f(\alpha)$ function depends on the nature of the process and is usually selected *a priori*. For the user's convenience, the notation of parameters and variables in Table SI.1 is the same as in Kinetics Neo software. Software uses the *p* parameter, which corresponds to the conversion, $p = \alpha$, and parameter $e = 1 - \alpha$ (the remaining fraction unreacted). The model-based analysis assumes that a reaction comprises several elementary reaction steps, which can be a series of consecutive reactions. In this approach, for each reaction step, kinetic triplicates and reaction type are constant throughout the reaction [3]. The reaction rate for individual reaction steps can be expressed through the Eq. (S3) [4]:

$$Reaction\ rate_j = A_j \cdot f_j(e_j, p_j) \cdot e^{-\frac{E_j}{RT}}, \tag{S3}$$

where $f_j(e_j,p_j)$ represents the function of reaction type, e_j is the initial reactant concentration, p_j is the product concentration, A_j is the pre-exponential factor, E_j is the activation energy, while j represents the number of specific reaction steps.

Table S1

Kinetic model functions (in differential form of the analytical kinetic functions, $f(\alpha)$) used in the current work, for computational procedure in the model-based (model-fitting) analysis.

**Model**	**Symbol**	$f(\alpha)$
Phase boundary-controlled reaction (contracting disk, 1D)	R1/F0	$(1 - \alpha)^0$
Phase boundary-controlled reaction (contracting area, 2D)	R2	$2\cdot(1 - \alpha)^{1/2}$
Phase boundary-controlled reaction (contracting volume, 3D)	R3	$3\cdot(1 - \alpha)^{2/3}$
Random nucleation, unimolecular decay law, first order chemical reaction	F1	$(1 - \alpha)$
Second order chemical reaction	F2	$(1 - \alpha)^2$
n-th order chemical reaction ($n \neq 1$)	Fn	$(1 - \alpha)^n$
Two-dimensional growth of nuclei (Avrami equation)	A2	$2\cdot(1 - \alpha)[- \ln(1 - \alpha)]^{1/2}$
Three-dimensional growth of nuclei (Avrami equation)	A3	$3\cdot(1 - \alpha)[- \ln(1 - \alpha)]^{2/3}$
n-dimensional nucleation (Avrami-Erofeev equation)	An	$n\cdot(1 - \alpha)[- \ln(1 - \alpha)]^{1-1/n}$
One-dimensional diffusion, parabola law	D1	$1/2\alpha$
Two-dimensional diffusion, Valensi equation	D2	$1/[- \ln(1 - \alpha)]$
Three-dimensional diffusion, Jander equation	D3	$(3/2)(1 - \alpha)^{2/3}/[1 - (1 - \alpha)^{1/3}]$
Three-dimensional diffusion, Ginstling-Brounstein	D4	$(3/2)/[(1 - \alpha)^{-1/3} - 1]$
Prout-Tompkins equation	B1	$(1 - \alpha)\cdot\alpha$
Expanded Prout-Tompkins equation	Bna	$(1 - \alpha)^n\cdot\alpha^a$
First order with autocatalysis	C1	$(1 + k_{cat}\cdot\alpha)(1 - \alpha)$
n-th order with autocatalysis	Cn	$(1 + k_{cat}\cdot\alpha)(1 - \alpha)^n$
n-th order and m-power with autocatalysis	Cnm	$(1 - \alpha)^n\cdot\alpha^m$
Expanded Šestak-Berggren (SB) equation	SBnmq	$(1 - \alpha)^n\cdot\alpha^m\cdot[-\ln(1 - \alpha)]^q$
Kamal-Sourour equation	KS	$(k_1 + k_2\cdot\alpha^m)(1 - \alpha)^n$
Nakamura crystallization	Nk (An + H-L)	$f(\alpha)\cdot K(T), f(\alpha) = n\cdot(1 - \alpha)[-\ln(1 - \alpha)]^{1-1/n}$, where for analytical dependence of the

		rate constant $K(T)$, Hoffman-Lauritzen (H-L) theory is used (non-Arrhenius).
Šestak-Berggren crystallization or Sbirrazzuoli crystallization	(SBC/SC) (SB + H-L)	$f(\alpha)\cdot K(T)$, $f(\alpha) = (1 - \alpha)^n\cdot\alpha^m\cdot[-\ln(1 - \alpha)]^q$, where for analytical dependence of the rate constant $K(T)$, Hoffman-Lauritzen (H-L) theory is used (non-Arrhenius)

The model-based analysis allows the addition of models *as per* their physical and chemical reactions of the material, whether it is a competitive step (parallel/split reaction) or a consecutive step (series reaction). For each reaction step, it is possible to add reaction types to determine kinetic triplicates, as well as optimize each reaction step with experimental results. After optimizing each reaction step, we can optimize the constructed model for the entire process of interest, for kinetic triplicates as well as for predictions [5]. The kinetics analysis can be used for predictions for measurement output (signals), conversion, conversion rate, concentration for each reactant (model-based), and then the reaction rate for each reaction step, depending upon the careful selection of experimental data fitting to a model-free or model-based analysis.

Usually, the reaction rate equation refers to the conversion value α, attached to fraction reacted, and therefore the above function $f_j(e_j,p_j)$ is usually expressed as $f(\alpha)$. The $f(\alpha)$ in Table SI.1 is the term that describes the dependence of the reaction rate with mechanism of the process. These mechanisms are proposed considering different geometrical assumptions for the shape of the material particles (spherical, cylindrical, and planar) and driving forces (interface growth, diffusion, nucleation and growth of nuclei). If the investigated process contains the reaction step sequence as **A** (1) → **B** (2) → **C** (3) → …, where a is the concentration of **A**, b is the concentration of **B**, c is the concentration of **C**, the rate-law equations take a form, such as: $\mathrm{d}(a \rightarrow b)/\mathrm{d}t = A_1 f_1(a)\exp(-E_{a1}/RT)$, $\mathrm{d}(b \rightarrow c)/\mathrm{d}t = A_2 f_2(b)\exp(-E_{a2}/RT)$, …, where A_1, A_2, E_{a1} and E_{a2} are the pre-exponential factors and activation energies, which correspond to appropriate consecutive reaction steps ((1), (2),…), while $f_1(a)$ and $f_2(b)$ are differential forms of the analytical kinetic functions (Table SI.1), related to the thermal transformation of the considered chemical species (compounds) (**A**, **B**, …). This example represents operational apparatus for model-based analysis which can involve considerations of complex process with several steps, i.e., the reaction steps are consecutive (or may be in parallel mode), where each reaction step has its own kinetic rate equation, while kinetic parameters of each considered step are constant values. It should be *noted* that all kinetic parameters such as activation energy, the pre-exponential factor, the order of reaction, and the reaction type are *assumed to be constant during the reaction progress*, for *every individual reaction step*.

Within the actual analysis, the thermo-analytical signal presented through the Eq. (S4):

$$m = m_o - \Delta m \cdot \left[\sum_{j=1}^{n} Contribution_j \int \left(\frac{d(x_j \rightarrow y_j)}{dt}\right)_j dt\right], \tag{S4}$$

is the sum of signals of the single reaction steps (m is the mass, m_o is the initial mass, Δm is the total mass change), where $(x_j - y_j)$ represents the heat flow on the reaction path from the reactant "x" to the product "y", while $Contribution_j$ corresponds to the contribution of the "j" reaction step to the overall heat flow. It should be said that

for single-step reactions, where the reaction model remains constant during the process, both, model-based and model-free approaches should provide results with the same or similar kinetic parameters values, which are fixed (for model-based analysis) or almost constant (for model-free analysis) in the reaction progression. For complex processes, where the kinetic mechanism is changing, a large difference may appear, in the interpretation of two methods results, provided by different approaches. For model-free methods, the change of the model is described by the continuous change of the kinetic parameters with the increase of the conversion. For model-based method, the change of the kinetic mechanism is simulated by several reaction steps, with own kinetic triplets (A_j, E_j, f_j), ranked in a specific reactions order, through a constructed mechanistic scheme, which has special marking in the computational software (kinetic scheme code designation), depending on whether the branching exists or not. Model-based kinetic analysis offers the possibility of visual design for kinetic models with an unlimited number of steps connecting in any combinations [6]. Model-based kinetic analysis is a powerful tool that allows accurate estimation of the number of steps, their contribution to the total effect of the reaction or about reaction order (or kinetic exponents) for each considered step. This analysis is based on the *assumption of a kinetic model of the process* (in this context, some *a priori* ideas can be used, concerning mechanisms of possible processes in the system under observation; this can be literature data, results of other physicochemical studies/experiments, or general considerations, based on theories of heterogeneous processes), which uses powerful mathematics to solve the system of differential equations, and make statistical comparison of the used models and therefore can answer all key mechanism issues, that may arise from the interpretation of the results of experimental measurements.

References

[1] J. Opfermann, Kinetic analysis using multivariate non-linear regression I. Basic concepts, *J. Therm. Anal. Calorim*. 60 (2000) 641-658. https://doi.org/10.1023/A:1010167626551.

[2] E. Moukhina, Determination of kinetic mechanisms for reactions measured with thermoanalytical instruments, J. *Therm. Anal. Calorim*. 109 (2012) 1203-1214. https://doi.org/10.1007/s10973-012-2406-3.

[3] E. Moukhina, Comparison of isothermal predictions based on model-free and model-based kinetic methods, *J. Test. Eval*. 42 (2014) 1377-1386. DOI:10.1520/JTE20140145.

[4] E. Moukhina, Initial kinetic parameters for the model-based kinetic method, *High. Temp. High. Press*. 42 (2013) 287-302.

[5] B. Janković, N. Manić, I. Radović, M. Janković, M. Rajačić, Model-free and model-based kinetics of the combustion process of low rank coals with high ash contents using TGA-DTG-DTA-MS and FTIR techniques, *Thermochim. Acta* 679(10) (2019) 178337. DOI:10.1016/j.tca.2019.178337.

[6] NETZSCH, Kinetics Neo Software, Product Version 2.6.0.1, Kinetic Analysis Software for Thermal Measurements of Chemical Reactions. Model-free and Model-based methods, 2022. https://kinetics.netzsch.com (accesed on 10 January 2023).

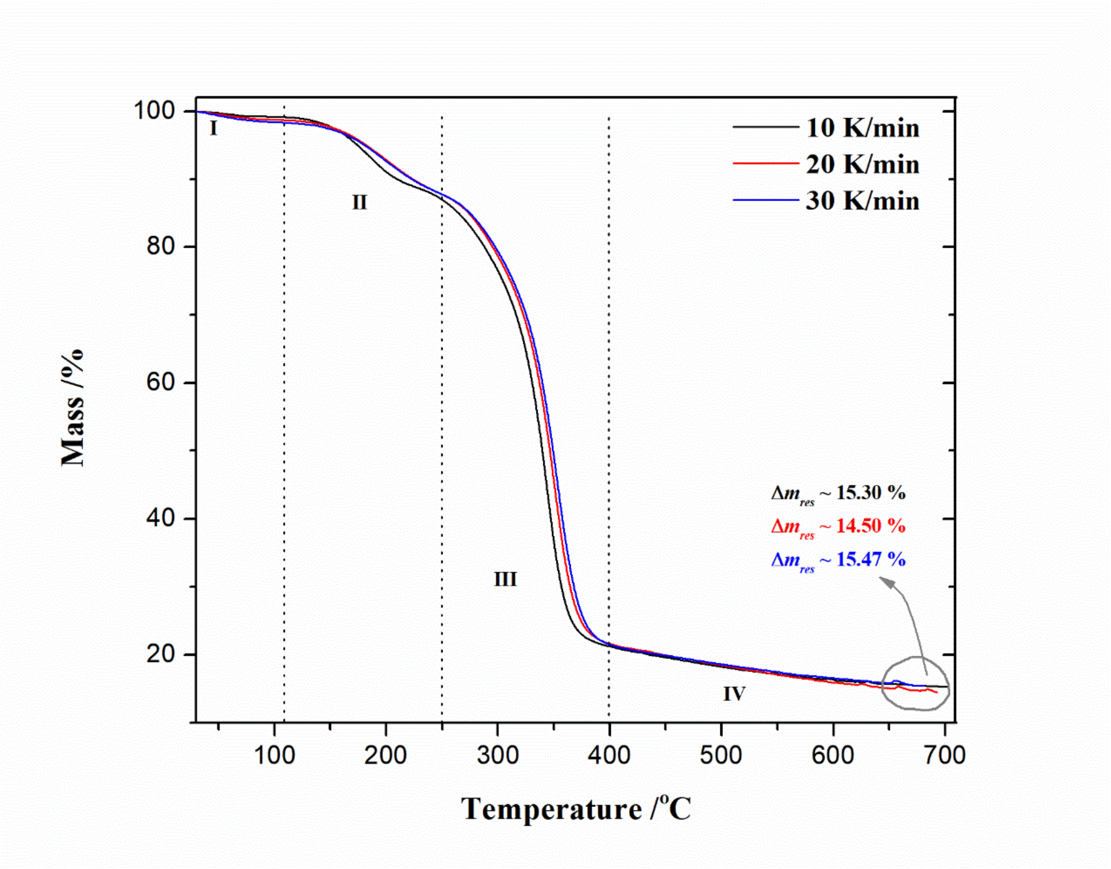

Figure S1. TG-curves at the various heating rates (*β* = 10, 20 and 30 K/min) in an argon (Ar) atmosphere, for devolatilization process of r-CAcF sample (designation of the main process stages ("I - IV") and corresponding residual mass values (Δm_{res}) are also presented).

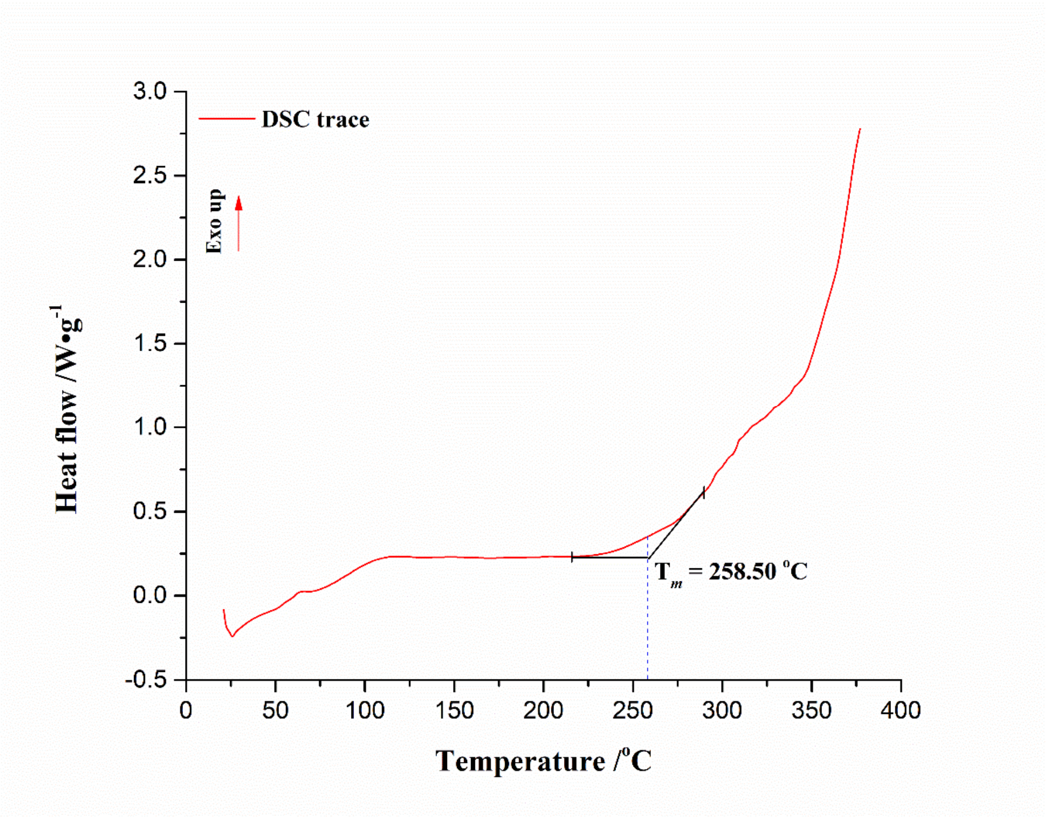

Figure S2. DSC curve of the r-CAcF sample at the heating rate of β = 10 K/min, measured in the temperature range of ΔT = +21 °C - +377 °C. The position of melting temperature (T_m) is indicated (T_m = 258.50 °C).

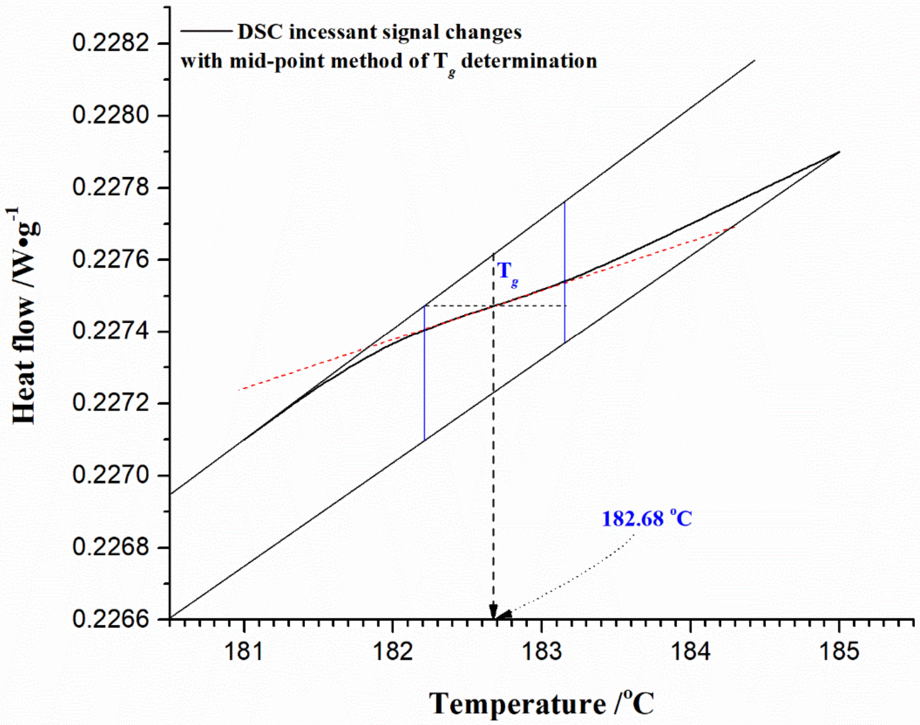

Figure S3. Determination procedure of the glass transition temperature (T_g) for r-CAcF sample.

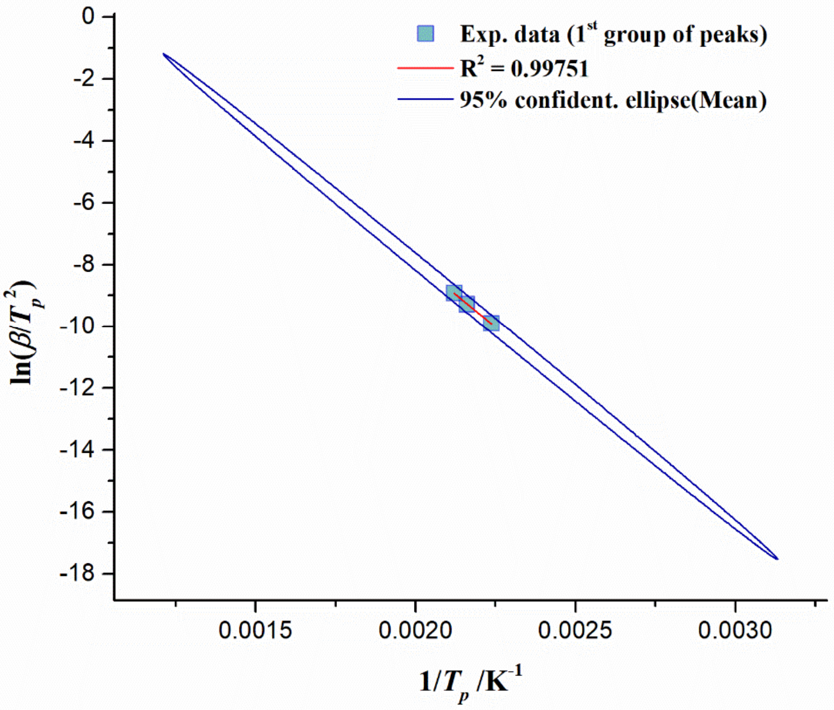

Figure S4. Kissinger plot for the thermal decomposition process of the r-CAcF sample, at different heating rates (β = 10, 20 and 30 K/min), including the 1st group of peaks in the Fig. 4 b) (Kinetic parameters values: E_a = 70.69 ± 0.30 kJ mol^{-1}; A = 1.27 × 10^6 s^{-1}).

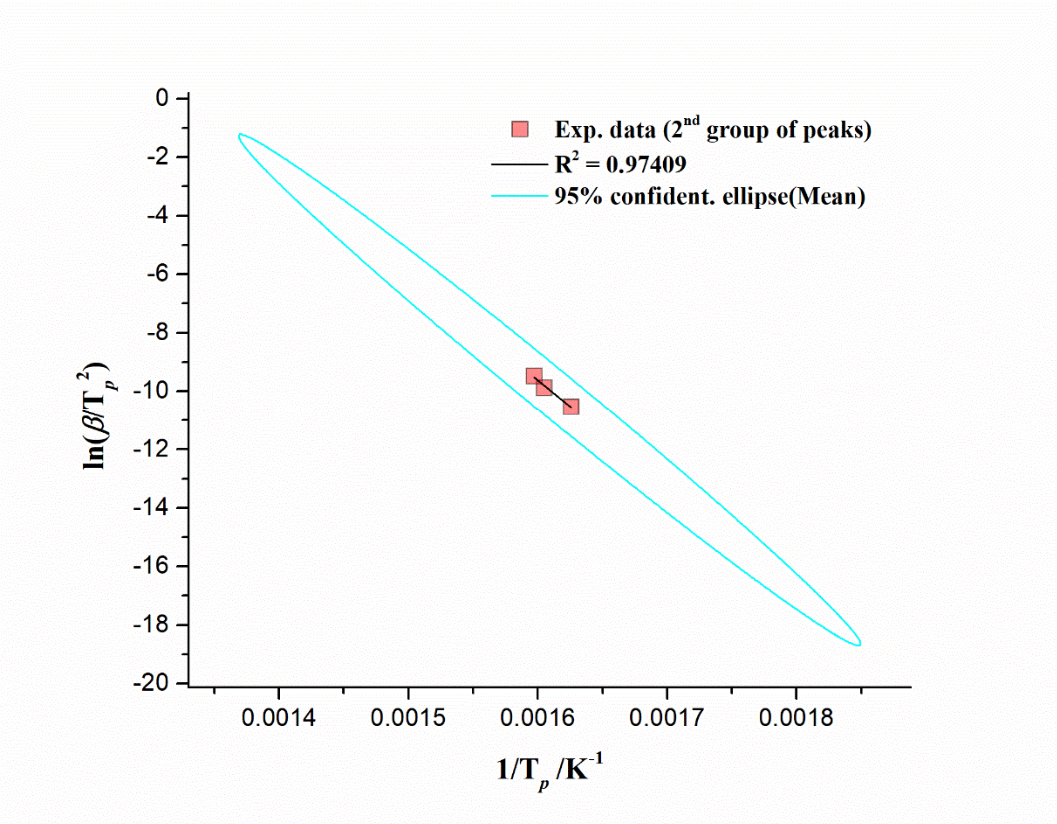

Figure S5. Kissinger plot for the thermal decomposition process of the r-CAcF sample, at different heating rates (β = 10, 20 and 30 K/min), including the 2nd group of peaks in the Fig. 4 b) (Kinetic parameters values: E_a = 300.34 ± 4.14 kJ mol^{-1}; A = 4.98 × 10^{23} s^{-1}).

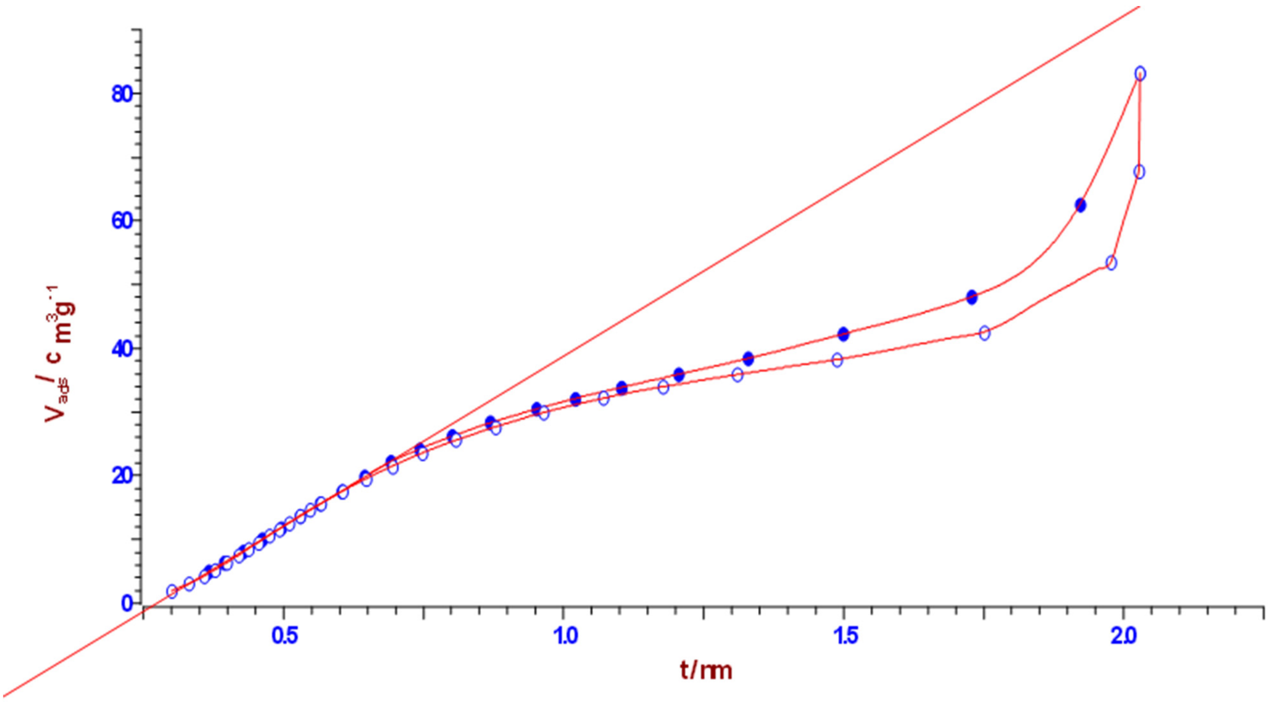

Figure S6. *t*-plot (Lippens and de Boer) method for determination of the micropore volume and external surface area, for CAc800(1h) sample (R^2 = 0.99868).
